# Identification and analysis of evolutionary selection pressures acting at the molecular level in five forkhead subfamilies

**DOI:** 10.1186/1471-2148-8-261

**Published:** 2008-09-24

**Authors:** Christina D Fetterman, Bruce Rannala, Michael A Walter

**Affiliations:** 1 Department of Medical Genetics, University of Alberta, Edmonton, Alberta, Canada; 2 Genome Center and Section of Evolution and Ecology, University of California, Davis, California, USA

## Abstract

**Background:**

Members of the forkhead gene family act as transcription regulators in biological processes including development and metabolism. The evolution of forkhead genes has not been widely examined and selection pressures at the molecular level influencing subfamily evolution and differentiation have not been explored. Here, *in silico *methods were used to examine selection pressures acting on the coding sequence of five multi-species FOX protein subfamily clusters; FoxA, FoxD, FoxI, FoxO and FoxP.

**Results:**

Application of site models, which estimate overall selection pressures on individual codons throughout the phylogeny, showed that the amino acid changes observed were either neutral or under negative selection. Branch-site models, which allow estimated selection pressures along specified lineages to vary as compared to the remaining phylogeny, identified positive selection along branches leading to the FoxA3 and Protostomia clades in the FoxA cluster and the branch leading to the FoxO3 clade in the FoxO cluster. Residues that may differentiate paralogs were identified in the FoxA and FoxO clusters and residues that differentiate orthologs were identified in the FoxA cluster. Neutral amino acid changes were identified in the forkhead domain of the FoxA, FoxD and FoxP clusters while positive selection was identified in the forkhead domain of the Protostomia lineage of the FoxA cluster. A series of residues under strong negative selection adjacent to the N- and C-termini of the forkhead domain were identified in all clusters analyzed suggesting a new method for refinement of domain boundaries. Extrapolation of domains among cluster members in conjunction with selection pressure information allowed prediction of residue function in the FoxA, FoxO and FoxP clusters and exclusion of known domain function in residues of the FoxA and FoxI clusters.

**Conclusion:**

Consideration of selection pressures observed in conjunction with known functional information allowed prediction of residue function and refinement of domain boundaries. Identification of residues that differentiate orthologs and paralogs provided insight into the development and functional consequences of paralogs and forkhead subfamily composition differences among species. Overall we found that after gene duplication of forkhead family members, rapid differentiation and subsequent fixation of amino acid changes through negative selection has occurred.

## Background

A highly conserved DNA binding domain, termed 'forkhead' due to the physical appearance of *Drosophila fork head *mutants, defines forkhead gene family members. Forkhead family members act as transcription activators or repressors in biological processes involved in development and metabolism. Human diseases such as Axenfeld-Rieger syndrome [1], lymphedema-distichiasis [2], developmental verbal dyspraxia [3], and various cancers [4-7] have been associated with mutations or chromosomal rearrangements of forkhead genes. Forkhead genes have been identified in a wide variety of animals and fungi but not plants. Within the forkhead gene family, subfamilies were delineated by their position within a phylogenetic tree that was created using only the forkhead domain sequences [8]. Different subfamilies are identified by letters, with subfamilies A through S noted in humans. For many species, multiple members of a subfamily are known to exist and are further delineated by Arabic numerals.

While some research has examined forkhead gene family evolution, selection pressures on individual codons have not been measured and studies that have examined evolutionary forces acting on entire forkhead genes have included only orthologous sequences from a subfamily. Here we analyze entire subfamilies to explore the evolutionary and functional significance of subfamily paralogs and orthologs. Gene duplication, and subsequent selection driving adaptive evolution, is thought to create gene families with differentiated family members. At the molecular level, amino acid changes that result in reduced fitness are removed by negative selection whereas changes that increase fitness are maintained by positive selection. When amino acid changes do not decrease or increase fitness, the changes are considered neutral. At individual codons, also known as sites, natural selection can be measured in terms of ω, the nonsynonymous substitution rate divided by the synonymous substitution rate. An ω < 1 indicates negative selection is occurring while ω > 1 suggests positive selection and ω = 1 for neutral changes. Negative or positive selection of amino acid residues implies that the residues are functionally important. Neutral changes at amino acid sites imply that the exact composition of amino acids at these sites is unimportant and that they are not directly involved in protein function.

We sought to identify the selection pressures acting on individual amino acid sites in forkhead gene family members. Five forkhead subfamilies, FoxA, FoxD, FoxI, FoxO and FoxP were examined independently using branch-site and site models implemented in the codeml program, contained in the PAML package. The results of our analysis of site and lineage specific selection patterns, in conjunction with prior information concerning the functional importance of amino acid residues in each cluster, provide insights into forkhead gene family evolution and information regarding potential functional and nonfunctional amino acids in this important transcription factor gene family.

## Methods

### Sequence Data

A list of 672 amino acid sequences containing the forkhead domain was retrieved from the NCBI Entrez Protein Database using the Conserved Domain Architecture Retrieval Tool (CDART) [9] in conjunction with the Conserved Domain Database forkhead domain definition, cd00059 [10,11]. Sequences described as partial, incomplete, fragment, predicted, putative and hypothetical as well as duplicates and isoforms were excluded resulting in a total of 299 sequences from 51 species analyzed. Initial analysis of all known forkhead genes simultaneously using global or local alignment methods, and parsimony, likelihood or Bayesian phylogenetic methods, produced trees with inconsistent subfamily placement due to low sequence homology outside of the forkhead domain among different subfamilies. BLASTCLUST was therefore used to cluster the amino acid sequences in groups of 30% identity over 90% of their length [12]. To improve selection analysis accuracy and power, only clusters containing 10 or more sequences were included in further analyses [13]. There were five clusters, named for the majority of the sequences contained within each one, chosen for further analysis: FoxA, FoxD, FoxI, FoxO and FoxP (see Additional file 1).

### Alignment and Phylogenetic Analysis

Each cluster was aligned independently using a combination of CLUSTALX1.83 [14] and CLUSTALW1.81 [15] (see Additional file 2). Amino acid sequences were aligned rather than nucleotide sequences so that gaps would not be introduced into the corresponding codons. The amino acid alignments were converted into nucleotide alignments, for phylogeny creation, utilizing the proteins' corresponding nucleotide sequences from GenBank with the program protal2dna2.0 [16]. The nucleotide alignment was then converted to nexus format with the ReadSeq2.93 [17] program for phylogenetic analysis.

MrModeltest2.2 [18] was used in conjunction with PAUP4.0b10 [19] to determine the best nucleotide substitution model for each cluster. The model chosen by the Akaike Information Criterion measure in MrModeltest was implemented in MrBayes3.1.1 [20] for each cluster. All priors were uninformative and set at default values. Each analysis was run for 1000000 generations, sampling every 100^th ^generation for a total of 10001 samples. A burn-in value, the number of initial samples removed from analysis, of 3000 was chosen based on previous analyses. The generation versus log probability plots were examined to ensure convergence was reached and that a burn-in of 3000 was appropriate. The potential scale reduction factor was also used as a measure of convergence [21].

### Identification of Selection Pressures

Values of ω were estimated for each non-ambiguous codon in the alignment using the codeml program contained in the PAML3.15 package [22]. Codon site models M0, M3, M1a, M2a, M7 and M8 that estimate ω, were implemented for each cluster [23-26]. Model M0 allows only one category of ω for all sites. Model M3 allowed three unconstrained ω categories, ω_1_, ω_2 _and ω_3 _with proportions p_1_, p_2 _and p_3 _= 1-p_1_-p_2_. Model M1a contains two categories of ω, 0 < ω_0 _< 1 and ω_1 _= 1 with proportions p_0 _and p_1 _= 1-p_0_. Model M2a adds a third category, ω_s _> 1 with proportion p_s _such that p_s _= 1-p_0_-p_1_. Models M7 and M8 both contain 10 equal proportion ω categories approximated from β(p, q) with 0< ω < 1 while Model M8 adds an additional ω category, ω_s _> 1. The proportion of sites with ω ~ β(p, q) is represented by p_0 _and those with ω_s _> 1 are represented by p_s _where p_s _= 1-p_0_. Each site is assigned to an ω category using a naïve empirical Bayes (NEB) (models M0, M3, M1a and M7) [27] or Bayes empirical Bayes (BEB) (models M2a and M8) [26] approach.

Codon frequencies were set as free parameters (CodonFreq = 3) and ambiguous columns in the alignment were removed from the analysis. The transition/transversion ratio and branch lengths were estimated from the data using maximum likelihood methods. Two separate analyses were conducted with initial values of 0.4 and 2.0 for ω to identify and avoid local optima [13,23]. Each analysis was repeated once. Comparison of the results for each model using ω = 0.4 and ω = 2 and their repeats revealed that parameter estimates (ln likelihood, p, ω and β(p, q)) for each model were identical when rounded to three decimal places. The accuracy and power of selection analysis are good if different models are tested, initial values of ω are varied and the analysis is consistent when repeated [23].

A likelihood ratio test (LRT) comparing M0 and M3 using a χ^2 ^distribution with four degrees of freedom was used as a test for variation in ω among sites [28,29]. Two LRTs were used as a test for positive selection, M1a against M2a and M7 against M8, each using a χ^2 ^distribution with two degrees of freedom [25,27]. The LRTs were considered significant when the P-value was ≤ 0.05. The critical values are 9.49 and 5.99 for four and two degrees of freedom respectively when P = 0.05. A correction for multiple tests was not performed as the two LRTs for positive selection test the fit of different distributions of ω to the data and are therefore performed for robustness [30].

If positive selection occurs in only a few lineages in a tree, it may not be identified using site models, therefore branch-site model A, which allows for ω > 1 along a specified lineage, the foreground branch, while ω cannot be greater than one in any of the other lineages, the background branches [31] was applied. This model was implemented for lineages leading to parologous clades in the FoxA, FoxD, FoxO and FoxP clusters as positive selection is a potential evolutionary force driving subfamily paralog functional differentiation. The FoxI cluster was not examined as no lineages of interest were identified. Model A contains four classes of sites; class 0: 0 < ω_0 _< 1 and class 1: ω_1 _= 1, with proportions p_0 _and p_1 _respectively, for both the foreground and background branches and class 2a or 2b: ω_2 _≥ 1 for the foreground branch with corresponding sites in the background lineage falling into class 2a: 0 < ω_0 _< 1 or class 2b: ω_1 _= 1 site classes with proportions (1-p_0_-p_1_)p_0_/(p_0_+p_1_) and (1-p_0_-p_1_)p_1_/(p_0_+p_1_) respectively. All other parameters and running conditions were set as described for the site models. Model A is compared to a null model A with ω_2 _= 1 fixed, using a LRT and χ^2 ^distribution with one degree of freedom. Statistical significance at α = 0.05 was determined after correction for multiple tests using Rom's procedure and the Bonferroni correction when multiple branches were tested in a phylogeny [32]. If significance was obtained through Rom's procedure but not the more stringent Bonferroni correction, the LRT was referred to as potentially positive. BEB is used to identify sites under positive selection if the LRT is significant and ω_2 _> 1.

### Identification of EH1 Motifs

The Engrailed Homology 1 (EH1) motif has previously been identified in many, but not all of the sequences included in this analysis [33,34]. Visual examination of the sequence alignments in conjunction with known EH1 locations suggested that there were EH1 motifs present in the sequences included in this analysis that have not been previously reported. A Perl script was written to search all of the sequences included in this analysis for the EH1 motif of the form XXaXbXXcdXX where X can be any amino acid, a can be Phe, His, Tyr or Trp, b and c can be Ile, Leu or Val and d can be Glu, Phe, His, Ile, Lys, Met, Gln, Arg, Trp or Tyr [33,35]. Sequences with newly identified EH1 motifs are indicated in Additional file 1 and the locations of the motifs can be found in Additional file 3(A-E).

## Results

### Branch-Site Analysis

**Figure 1:**
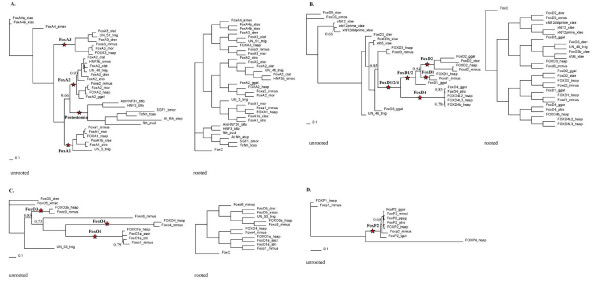
**Branches tested for positive selection using branch-site models.** In each phylogeny the branches tested are indicated with red stars and labels representing the clade of interest. The trees in which positive selection was identified were rooted using a set of FoxC genes as an outgroup in conjunction with neighbor joining tree creation, after branch-site analysis for clarity into evolutionary relationships. Clade credibility values less than 0.95 are indicated on the unrooted trees. The phylogenies are for the gene clusters as follows; **A. **FoxA **B. **FoxD **C. **FoxO **D. **FoxP.

Figure 1 shows the branches that were tested for positive selection in each of the gene clusters. LRTs (Table 1) were significant for branches leading to the FoxA3 and Protostomia clades in the FoxA cluster and the FoxD2 lineage in the FoxD cluster and potentially significant for the FoxD1/2/4 lineage in the FoxD cluster and the FoxO3 lineage in the FoxO cluster, suggesting that positive selection has acted in the diversification of these paralogs from other genes in the cluster. Model A parameter estimates for lineages under positive selection are given in Table 2. Positive selection was not identified in any of the other lineages tested.

**Table 1:** Statistical significance of the branch-site analysis LRTs after multiple corrections using Rom's procedure and the Bonferroni correction.

Cluster	Lineage Tested	p-value of LRT	Rom's Procedure critical value	Bonferroni critical value
FoxA	FoxA1	0.62	0.0127	0.0125
	FoxA2	0.0634	0.0169	0.0125
	FoxA3	0.0032	**0.025**	**0.0125**
	Protostomia	< 0.0001	**0.05**	**0.0125**

FoxD	FoxD1	1	0.0102	0.01
	FoxD4	0.0973	0.0127	0.01
	FoxD1/2	0.0347	0.0169	0.01
	FoxD1/2/4	0.0234	**0.025**	0.01
	FoxD2	< 0.0001	**0.05**	**0.01**

FoxO	FoxO4	0.2628	0.0169	0.0167
	FoxO1	0.109	0.025	0.0167
	FoxO3	0.0177	**0.05**	0.0167

FoxP	FoxP2	1	0.05	0.05

Critical values of statistically significant results are in boldface.

**Table 2:** Model A parameter estimates for significant branch-site LRTs.

Cluster	Lineage	Site Class	Proportion	Backgound ω	Foreground ω	Positively Selected Sites* (P ≥ 0.95)
FoxA	FoxA3	0	0.63948	0.02356	0.02356	FOXA3_hsap 27P, 36G, 112P, 113L
		1	0.330014	1	1	
		2a	0.04109	0.02356	999	
		2b	0.01929	1	999	
	
	Protostomia	0	0.62704	0.02362	0.02362	FOXA1_hsap 68Y, 159A, 199W, 234S, 237K, 242S
		1	0.29309	1	1	
		2a	0.05443	0.02362	999	
		2b	0.02554	1	999	

FoxD	FoxD1/2/4	0	0.53693	0.02506	0.02506	FOXD2_hsap R390
		1	0.41932	1	1	
		2a	0.0456	0.02506	71.85587	
		2b	0.01918	1	71.85587	
	
	FoxD2	0	0.51695	0.02526	0.02526	FOXD2_hsap E242, T386, L389, R390, Q391, G392, L393, K394, T395
		1	0.36815	1	1	
		2a	0.06711	0.02526	999	
		2b	0.04779	1	999	

FoxO	FoxO3	0	0.80461	0.04277	0.04277	FOXO3_hsap S280
		1	0.14422	1	1	
		2a	0.04339	0.04277	10.7599	
		2b	0.00778	1	10.7599	

* The sequence to which amino acid residues reported correspond is given for each lineage.

In the FoxD2 clade one positively selected site occurs between the forkhead domain and the EH1 motif in a region that has not been functionally characterized while the remaining positively selected sites identified in this lineage and that identified in the FoxD1/2/4 lineage occur within the EH1 motif as identified in the FoxD1, FoxD3 and FoxD5 sequences (see Additional file 3(B)). The LRT for the FoxD1/2/4 branch was potentially significant and the amino acid residues at the positively selected site identified in the FoxD1/2/4 lineage differ only in the FoxD2 lineage and are otherwise 100 percent conserved in the other sequences analyzed, therefore it is unlikely that positive selection acted along the FoxD1/2/4 lineage. The FoxD2 lineage sequences contain an EH1 motif however it was not aligned with that identified in the FoxD1, FoxD3 or FoxD5 sequences due to additional amino acids, some of which were under positive selection, found in the FoxD2 lineage. It is likely that the positive selection identified in the FoxD2 lineage within this region is due to the high conservation of the EH1 motif in the other sequences analyzed and lack of motif alignment and not due to evolutionary forces.

### Site Analysis

**Figure 2:**
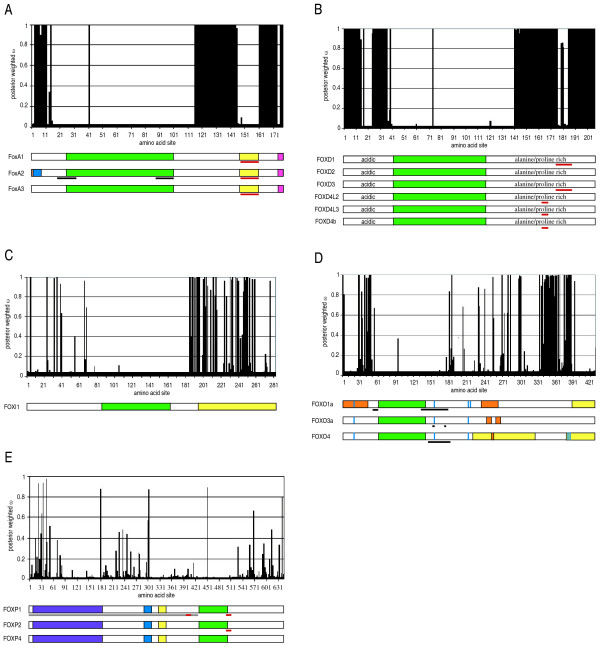
**Selection pressures on amino acids in each of the six clusters analyzed.** In each cluster the forkhead domain position was obtained from the NCBI Entrez Protein [11] database record for that sequence (see Additional file 1 for accession numbers). **A. **FoxA cluster of 31 sequences. Peach: conserved domain IV, site 1 [36,74] Blue: conserved domain V, sites 2–7 [36,74] Green: forkhead domain, sites 25–101 Yellow: conserved domain II, sites 147–160 [72] Pink: conserved domain III, sites 174–177 [72] Black Bar: nuclear localization signal, sites 19–32 and 88–101 [74] Red Bar: EH1 motif, sites 151–160 [33,34]**B. **FoxD cluster of 24 sequences. Green: forkhead domain, sites 42–119 Acidic and alanine/proline rich regions described by Ernstsson et al. 1996; Sutton et al. 1996; Ernstsson et al. 1997; Freyaldenhoven, Fried, and Wielckens 2002. Red Bar: EH1 motif, sites 163–169 for all of the FOXD4s, sites 176–186 for FOXD1 and FOXD3 [33,34]**C. **FoxI cluster of 10 sequences. Green: forkhead domain, sites 123–200 Yellow: transactivation domain, sites 196–282 [78]**D. **FoxO cluster of 12 sequences. Green: forkhead domain, sites 61–141 Orange: nuclear export signal, sites 1–43 and 235–264 for FOXO1a, sites 244–252 and 258–266 for FOXO3a, sites 250–256 for FOXO4 [42,46,47,76] Black Bar: nuclear localization signal, sites 52–60 and 134–180 for FOXO1a, sites 152–154 and 173,174 for FOXO3a, sites 144–183 for FOXO4 [42,45,46] Yellow: transactivation domain, sites 389–428 for FOXO1a, sites 221–326 and 378–428 for FOXO4 [38,39] Blue Bar: phosphorylation site, sites 20, 157 and 216 for FOXO1a, FOXO3a and FOXO4, additionally site 218 for FOXO1a, sites 379 and 383 for FOXO4 [44,48-52,55,56]**E. **FoxP cluster of 10 sequences. Purple: glutamine rich region, sites 6–182 [58,61,79-81] Blue: zinc finger, sites 288–311 [58,60,61,81] Yellow: leucine zipper, sites 324–349 [58-61] Green: forkhead domain, sites 434–506 Grey Bar: region involved in repression, sites 1–505 [59,80] Red Bar: EH1 motif, sites 398–408 and 501–511 for FOXP1, sites 501–511 for FOXP2.

Codon site models M0, M1a, M2a, M3, M7 and M8 were implemented in codeml for each of the six clusters and compared using likelihood ratio tests. For each cluster the M3 vs. M0 LRT was significant (Table 3), indicating that one category of ω was insufficient to describe the variability in selection pressure across amino acid sites. LRTs testing for positive selection, M2a vs M1a and M8 vs M7, were also insignificant for each cluster (Table 3), therefore the amino acid changes within each cluster are neutral or under negative selection. Table 4 reports the parameter estimates for the least parameter rich model, M1a, which best describes the variation in selection pressures across sites. Graphs were constructed showing the posterior weighted ω, the mean of ω over the site classes weighted by the posterior probability of each class, of each residue analyzed (Figure 2). Since ambiguous sites were removed, the residue numbers along the bottom of the graphs do not correspond to residue numbers of the analyzed sequences. Underneath each graph is a cartoon of the important regions contained in human forkhead gene(s) within that cluster. Few functional regions have been examined in human FoxA and FoxP proteins therefore functional information identified in rat and mouse protein studies has been included in the FoxA and FoxP figures respectively. The location of the forkhead domain for each human sequence was taken from the NCBI Entrez Protein [11] database record for that sequence.

**Table 3:** Site analysis LRT results for each cluster.

	Models Compared					
	
	M3 vs. M0		M2a vs. M1a		M8 vs. M7	
**Cluster**	**2(ln_M3_-ln_M0_)**	**P-value**	**2(ln_M2a_-ln_M1a_)**	**P-value**	**2(ln_M8_-ln_M7_)**	**P-value**
FoxA	1446.504	** < 0.0001**	0	1	0	1
FoxD	1252.774	** < 0.0001**	0	1	0	1
FoxI	649.137	** < 0.0001**	0	1	0	1
FoxO	687.901	** < 0.0001**	0	1	0	1
FoxP	135.938	** < 0.0001**	0	1	0	1

Statistically significant results at α = 0.05 are in boldface.

**Table 4:** Parameter estimates of site model M1a for each cluster.

Cluster	Model	Parameter Estimates
FoxA	M1a	ω_0 _= 0.024 ω_1 _= 1 p_0 _= 0.682 p_1 _= 0.318
FoxD	M1a	ω_0 _= 0.025 ω_1 _= 1 p_0 _= 0.555 p_1 _= 0.445
FoxI	M1a	ω_0 _= 0.039 ω_1 _= 1 p_0 _= 0.832 p_1 _= 0.168
FoxO	M1a	ω_0 _= 0.042 ω_1 _= 1 p_0 _= 0.839 p_1 _= 0.161
FoxP	M1a	ω_0 _= 0.019 ω_1 _= 1 p_0 _= 0.970 p_1 _= 0.030

## Discussion

### Prediction of Functional and Nonfunctional Residues Using Site Analysis

The site methods described in this paper may be used to predict functionally important residues in gene family members. If a functional domain has been identified in one member of a gene family, but not in a different member and the functional domain is under strong negative selection, prediction of a similarly functioning domain may be made in the family member where a domain has not been identified. In support of this theory, the forkhead domain, which is most likely functionally active in all of the sequences analyzed, was under strong negative selection in each cluster. We were able to predict functional domains in the FoxA, FoxO and FoxP cluster sequences.

In the FoxA cluster conserved domain II has been shown to be involved in transactivation [36] and repression [37] in rat FoxA2. Since conserved domain II is entirely under strong negative selection (Figure 2A) and contained only one ambiguous column in the alignment (see Additional file 3(A)), it is likely functionally important in all of the sequences analyzed. In the FoxO cluster, a transactivation domain has been identified at the C-terminus of FOXO1a and FOXO4 [38,39] while a transactivation domain has yet to be identified in FOXO3a. A portion of the C-terminal transactivation domain in FOXO4 and the entire transactivation domain in FOXO1a was under strong negative selection (Figure 2D), therefore a C-terminal transactivation domain consisting of the negatively selected residues (sites 389–428 in Figure 2D, residues 605–673 in FOXO3a) may be predicted in FOXO3a. A second, weaker, transactivation domain was identified in FOXO4 between the forkhead domain and the C-terminal transactivation domain [38]. This region is not highly conserved, although small islands of consecutive columns without gaps in the alignment that show strong negative selection, i.e. sites 315–326 in Figure 2D, may be functionally important. C-terminal deletions of PAX3-FOXO1a (a fusion protein consisting of the PAX3 N-terminal region, which includes two DNA binding domains, to the C-terminal region of FOXO1a, that includes part of the forkhead domain and the C-terminal transactivation domain) that include residues within FOXO1a corresponding to the FOXO4 transactivation domain have also shown reduced transactivation [40,41]. The residues under negative selection in this region may be key to the transactivation function seen in FOXO1a and FOXO4, and residues of FOXO3a within this region may also show transactivation function. A N-terminal NES and a NLS at the N-terminus of the forkhead domain have been identified in FOXO1a [42] and were found to be under strong negative selection (Figure 2D). These regions have not been examined for NES or NLS function in FOXO3a and FOXO4. The strong negative selection of these regions suggests that a NES may be found in the N-terminus and an NLS at the N-terminus of the forkhead domain in all of the sequences analyzed. Similarly, three phosphorylation sites involved in cellular localization have been identified in FOXO1a, Ser322, Ser325 and Ser329 and have not been examined in FOXO3a and FOXO4 [43,44]. The Foxo6_mmus sequence was the only sequence that did not contain serines at these three positions (see Additional file 3(D)) suggesting that these serines may be functionally important in the other sequences analyzed with the exception of Foxo6_mmus. Broadly defined NLSs have also been described C-terminal to the forkhead domain in FOXO1a [45] and FOXO4 [46]. A NLS has not been defined in FOXO3a, however residues Arg248ArgArg and Lys269LysLys have been shown to function in nuclear localization [47]. This region is under strong negative selection, with the exception of one site, 181 in Figure 2D, which is under very weak negative selection, suggesting that a NLS may be found at this point in all of the sequences analyzed. Finally, there are three common phosphorylation sites among the FOXO proteins (sites 20, 157 and 216 in Figure 2D) and two 14-3-3 protein binding sites (sites 17–22 and 153–159 in Figure 2D) that are important in cytoplasmic/nuclear localization and therefore transactivation activity [42,45-57]. These phosphorylation and 14-3-3 binding sites were are all highly conserved among species and under strong negative selection suggesting functional importance in all of the sequences analyzed. Within the FoxP cluster the leucine zipper and zinc finger identified in FOXP1 and mouse Foxp1, Foxp2 and Foxp4 [58-61] were under strong negative selection suggesting that they are present in the other sequences analyzed (Figure 2E). The leucine zipper allows FoxP proteins to form homo- and hetero-dimers [59,60] and although the zinc finger function has yet to be determined, it has been suggested that it aids in dimer formation [60].

Additionally, functional domains may be predicted in regions under strong negative selection where a domain is not known to exist. For example, functionally important residues have not been identified in the N-terminus of FOXD proteins and a series of amino acids under strong negative selection is found in this region (Figure 2B). This series of negatively selected amino acids may be functionally important and forms a starting point to identifying functionally important residues outside of the forkhead domain in the FOXD proteins. Predicting functionally important residues with these methods provides a specific region of amino acids and potential domain boundaries that should be tested when searching for functional domains *in vitro*.

When a functional region has been identified in one gene family member, but the majority of the amino acids making up the functional region are aligned with gaps and/or are experiencing neutral changes, the region is likely not functioning in the same manner in the other sequences analyzed. Examples include conserved domains IV and V in the FoxA cluster and the transactivation domain in the FoxI cluster (Figure 2A, C, see Additional file 3(A, C)). This method identifies a region of amino acids that are less likely to be important for a specific function, which may then be examined last for functional significance when using *in vitro *methods.

### Refining Domain Boundaries Using Site Analysis

Domain boundaries are often identified by sequence comparison to functionally related proteins or through mutagenesis experiments. When comparing sequences, it is assumed that the domain boundaries are accurately defined in the protein to which the comparison is made. Often, the boundaries of a new domain are loosely defined through mutagenesis experiments, as it is too time consuming to examine every amino acid near the suspected boundary for functional contribution. These loosely defined domains are then used by other researches in sequence comparisons to identify domains in related proteins. The methods used in this paper provide a new *in silico *procedure for identifying domain boundaries. For example, residues 1–50 of FOXO1a have been identified as a NES [42] however, only residues 8–32 were under strong negative selection. This suggests that the functional domain boundaries of the N-terminal NES in FOXO1a may be redefined from residues 1 and 50 to residues 8 and 32. Molecular analysis is necessary to confirm the reallocation of domain boundaries.

The assigned boundaries of the forkhead domain vary from source to source. The NCBI Conserved Domain Database (CDD) definition of the forkhead domain, which was taken from the SMART database forkhead definition, was used in this paper. In this definition, the boundaries of the forkhead domain are defined by tertiary structure and sequence comparison of all known forkhead domains [62]. Since the C-terminal end of the forkhead domain is unstructured and variable among subfamilies [63-67], this region is excluded from the CDD forkhead domain definition even though it is involved in DNA binding [68-70]. When a new protein containing a forkhead domain is described in the literature, the forkhead domain is often identified through sequence comparison to the rat FoxA1 forkhead domain, the first forkhead domain containing protein identified in mammals [71]. The rat FoxA1 forkhead domain was broadly defined through mutational analysis [71] and then succinctly defined through sequence comparison to the rat FoxA2, FoxA3 and *Drosophila *Fork Head proteins [72,73]. When a forkhead domain is defined through sequence comparison to rat FoxA1, the N- and C-terminal domain boundaries vary within the gene family and subfamilies while the CDD definition of the forkhead domain is consistent among gene family members. The N- and C-terminal domain boundaries include additional amino acids when defined through sequence comparison to rat FoxA1 as compared to the CDD definition. In this analysis, a series of residues directly adjacent to the N- and C-termini of the forkhead domain in each of the clusters analyzed (Figure 2) were under strong negative selection, suggesting that the forkhead domain definition should include these residues. The forkhead domain definitions supplied in the literature often accounted for some of the negatively selected sites not included in the CDD forkhead definition; however, the literature definitions either included sites that were not conserved among species, included sites with neutral changes, did not include all of the sites under negative selection and all varied in their start and stop points within subfamilies. If the N- and C-terminal boundaries of a domain are defined as the first and last residue respectively of a series of residues under strong negative selection, the results will be reproducible and consistent among gene family or subfamily members.

### Identification of Amino Acids Involved in Paralog or Ortholog Differentiation

The branch-site and site analysis of selection pressures on codons conducted here have identified specific amino acids responsible for differentiation of paralogs in the FoxA and FoxO clusters and orthologs in the FoxA cluster. In the FoxA cluster, the region N-terminal to the forkhead domain appears to contribute to paralog differentiation. One positively selected site identified in the FoxA3 clade occurs within conserved domain IV and one positively selected site identified in the Protostomia lineage occurs within conserved domain V as both domains are defined in FoxA2 [74] (see Additional file 3(A)). Overall conserved domains IV and V, which have been shown to play a role in transactivation in FoxA2 proteins [74], are not well conserved in the FoxA3 or Protostomia proteins as compared to the FoxA1 and FoxA2 proteins as the majority of the residues making up these domains were not analyzed due to gaps in the alignment and those that were examined by site analysis show variability in selection pressure with most of the sites, 5/7, having experienced neutral changes (Figure 2A). Additional sites under positive selection N-terminal to the forkhead domain were also identified through branch-site analysis in the FoxA3 and Protostomia lineages (see Additional file 3(A)). Two of these sites in the FoxA3 lineage occur in a nuclear localization signal (NLS) that was broadly defined in rat FoxA2 [74] while the other positively selected sites are found in regions uncharacterized in any FoxA protein. FoxA1 and FoxA2 have more similar expression patterns and functions during development and metabolism as compared to the FoxA3 proteins (reviewed by [75]). This evidence in conjunction with the positive selection identified here suggests that the N-terminal region of sequences not included in the FoxA1 or FoxA2 clades have evolved to differentiate these proteins from the FoxA1 and FoxA2 proteins while the sequences were conserved in the FoxA1 and FoxA2 proteins leading to overlapping expression and function.

Conserved domain III, which has been shown to function in transactivation in rat FoxA2 [36] contained many ambiguous sites in the FoxA alignment (see Additional file 3(A)) due to sequences from the Protostomia lineage and variations in selection pressure were observed in the four sites, through site analysis, that did contain amino acids from these species (Figure 2A). This suggests that conserved domain III is important for FoxA function in the Deuterostomia but not in the Protostomia and that the FoxA genes in the two lineages have evolved to perform species specific functions. Therefore the presence of conserved domain III may differentiate FoxA orthologs between the Protostomia and Deuterostomia lineages.

In the FoxO cluster, the NES(s) located between the forkhead domain and the C-terminus in the FOXO1a, FOXO3a and FOXO4 sequences [42,46,47,76] are not highly conserved among the FoxO family members as their alignment was not well defined, only three sites, 250–252, in Figure 2D contain NES residues from each of the three human FOXO proteins examined and some residues have experienced neutral changes as demonstrated by site analysis. These NES(s) may be used to differentiate FoxO paralogs.

Only one site was found to be under positive selection in the FoxO3 lineage during branch-site analysis and the LRT was potentially significant. This residue is found in a region important for nuclear localization, C-terminal to the forkhead domain (see Additional file 3(C)). The amino acid located at the positively selected site is serine in the FoxO3 sequences while it is glycine, alanine or aspartic acid in the other sequences analyzed. The presence of serine at this position may be important for regulation of the FoxO3 proteins by phosphorylation and this regulation may be different from the other FoxO sequences analyzed. Molecular testing is required to validate this hypothesis.

In summary, residues that differentiate paralogs were identified in the FoxA and FoxO clusters while residues that differentiate orthologs were also identified in the FoxA cluster. This information provided insights into the evolution of these two subfamilies. Within the FoxD, FoxI, and FoxP clusters, residues that differentiate orthologs or paralogs were unidentifiable due to lack of functional information (FoxD and FoxI clusters only) and overall negative selection in the identified domains.

### Subfamily Evolution

Forkhead subfamilies are defined by their homology in the forkhead domain alone. Here we analyzed the entire coding regions of forkhead proteins and found that the subfamily structures were maintained after sequence analysis with BLASTCLUST. Our site analysis also demonstrated distinct regions of homology outside the forkhead domain in each of the clusters analyzed, further supporting the subfamily member evolutionary relationships defined by the forkhead domain alone.

The patterns of strong negative and neutral selection observed through site analysis in each of the clusters and through branch-site analysis along the majority of the lineages tested, indicate that after gene duplication, rapid differentiation of paralogs through codon changes and subsequent maintenance, negative selection, of these changes has occurred. The lack of positive selection observed through site analysis indicates that the functions of forkhead gene family members as we see them today have been determined and fixed in the species analyzed. However, the positive selection observed along select lineages in the FoxA and FoxO cluster indicate more recent or observable continuing functional divergence. While the majority of studies that have used these methods focus only on positive selection, a few involving transcription factor gene families have discussed negative selection as well. Our results are similar to those seen in a comparable analysis of *HOX7 *where heterogeneous selection pressures but not positive selection were observed during site analysis and positive selection was observed on a single branch separating paralogs during branch-site analysis [77]. These types of analysis of gene families that were originally defined by a common functional motif may confirm or refute the family relationships and provide insights into their evolutionary development. If positive selection is observed it suggests that the evolutionary changes are ortholog or paralog differentiating while negative selection indicates that the protein function is conserved among species.

### Forkhead Domain Evolution

As forkhead subfamilies are defined by and forkhead gene function is reliant on the forkhead domain, identification of selection pressures acting on codons within the domain provides insights into the functional evolution of subfamilies and their paralogs. In each of the subfamilies, the majority of the residues in the forkhead domain were under strong negative selection (Figure 2) consistent with the general consensus that the domain is highly conserved and important for proper gene function. More interestingly, sites under positive selection and neutral changes were observed in the forkhead domain in some subfamilies and these provide insights into the evolutionary differentiation of forkhead genes.

**Figure 3:**
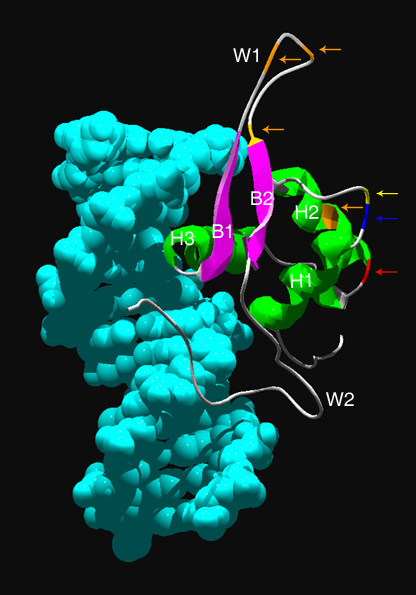
**Residues experiencing neutral changes and positive selection in the forkhead domain.** The forkhead domain of FOXA3 is shown bound to DNA. Residues with neutral changes identified in the FoxA (blue), FoxD (red) and FoxP (yellow) clusters and residues under positive selection identified in the Protostomia lineage of the FoxA cluster (orange) are highlighted and indicated with arrows. Alpha helices 1, 2 and 3, beta sheets 1 and 2, and wings 1 and 2 are denoted H1, H2, H3, B1, B2, W1 and W2 respectively.

In the FoxA cluster Protostomia lineage a number of residues under positive selection were found in the forkhead domain through branch-site analysis. These residues are located within helix 2, β-sheet 2 and wing 1 as defined by the crystal structure of FoxA3 [63] (Figure 3, see Additional file 3(A)). The residues corresponding to the positively selected sites in the Protostomia lineage are 100 percent conserved among the other sequences analyzed. It is possible that these changes in amino acid composition of the forkhead domain alter the domain configuration thus allowing for different target binding and/or regulation of FoxA genes in the Protostomia as compared to the Deuterostomia. It is interesting to note that to date, in most Protostomia only one FoxA class gene has been identified while in the Deuterostomia, multiple FoxA class genes have been found. If FoxA targets are similar in the Protostomia and Deuterostomia lineages, the alterations in the forkhead domain of Protostomia FoxA may allow these single proteins to perform the same function that require multiple FoxA proteins in the Deuterostomia. This theory is further supported by the differences observed in the N-terminal region of the Protostomia FoxA and in conserved domain III as compared to the Deuterostomia discussed earlier.

One residue within the forkhead domain was experiencing neutral changes in the FoxA, FoxD and FoxP clusters (Figures 2A (site 41), 2B (site 74), 2E (site 451)). The locations of the residues with neutral changes are shown on the FoxA3 crystal structure in Figure 3. The sites experiencing neutral changes identified in the FoxA and FoxP clusters were found at the C-terminus of alpha helix 1 while the site experiencing neutral changes in the FoxD cluster was located near the C-terminus of alpha helix 2. Neutral changes at a site imply that any amino acid may be present at that site and amino acid changes will not affect protein function. In support of this theory, mutation of the site corresponding to the neutral site identified in the FoxD cluster in rat FoxA3 from aspartate to lysine did not affect DNA binding [68]. The sites with neutral changes identified in the FoxA, FoxD and FoxP clusters and the corresponding sites in other Fox proteins have not been associated with point mutations causing human disease and have not been shown to contact DNA during DNA binding. The NCBI Entrez SNP database [11], Build 126, was used to determine if the sites with neutral changes have naturally occurring single nucleotide polymorphisms in any of the forkhead genes found in humans. Only one forkhead gene, FOXD4, has a known SNP at a location corresponding to one of the sites with neutral changes. The SNP identified in FOXD4 corresponds to the neutrally changed site identified in the FoxD proteins and is either aspartate or glycine. It would be interesting to determine if amino acid changes at these sites affect forkhead domain function and if the neutrally changed sites are common to the forkhead domain or specific to the subfamilies in which they were identified.

The variations from negative selection in the forkhead domain identified here may account for differences in subfamily and paralog function that are not explained by differences in timing or location of expression or other functional regions in the proteins.

## Conclusion

This analysis has provided insights into forkhead gene family and subfamily evolution. Through identification of selection pressures we provided evidence for the functional and evolutionary importance of amino acid differences in paralogs and orthologs of FOX subfamilies. Our work has also supported the forkhead subfamily structure and identified a pattern of evolution in the family. Additionally, our analyses allowed evaluation and extension of domain structural and positional information between gene family members. Future *in vitro *studies may use this information as a starting point or for refinement of protein functional analysis.

## Authors' contributions

CF participated in study design, carried out all experiments and drafted the manuscript. BR and MW conceived of the study and participated in its design. MW assisted in manuscript preparation.

## Supplementary Material

Additional file 1 Composition of the sequence clusters analyzed. This table gives the sequence composition of the clusters analyzed and notes sequences in which EH1 motifs were newly identified. Click here for file

Additional file 2 Alignment procedure with ClustalX and ClustalW. The procedure used to create multiple sequence alignments is provided in this file. Click here for file

Additional file 3 Amino acid alignments. The amino acid alignment of each of the clusters analyzed (A. FoxA, B. FoxD, C. FoxI, D. FoxO and E. FoxP) with regions of interest highlighted is shown here. Click here for file
