# Supplementary material for: Identification and analysis of evolutionary selection pressures acting at the molecular level in five forkhead subfamilies
Source: BMC Evol Biol. 2008 Sep 24;8:261. doi: 10.1186/1471-2148-8-261 (PMC2570691; doi:10.1186/1471-2148-8-261)
Supplement: Additional file 1 — Composition of the sequence clusters analyzed. This table gives the sequence composition of the clusters analyzed and notes sequences in which EH1 motifs were newly identified. [file 1471-2148-8-261-S1.pdf]

Additional file 1

Composition of the sequence clusters analyzed.

The table shows the composition of the five clusters of sequences with 30% identity over 90% of their length that were analyzed. The number of sequences in each cluster is given beside the cluster name. All sequences, excluding those indicated by *, also grouped in the given clusters at 40% identity over 90% of their length. Sequences in which EH1 motifs were newly identified are indicated by Φ. Protein and nucleotide accession numbers are from the NCBI Entrez Protein and Nucleotide databases respectively.

FoxA Cluster 31 sequences

Species	Sequence Identifier	Protein Accession #	Nucleotide Accession #
Achaearanea tepidariorum	At.fkh_atep Φ	BAC24088	AB096073.1
Ambystoma mexicanum	FoxA4_amex	AAC60128	U43547.1
Bombyx mori	SGF1_bmor	Q17241	D38514
Branchiostoma floridae	AmHNF31_bflo	CAA65368	X96519.1
Branchiostoma floridae	HNF3_bflo Φ	CAA70438	Y09236.1
Colisa lalia	FoxA2_clal Φ	BAB21570	AB050937.1
Danio rerio	FoxA2_drer	NP_571024	NM_130949.1
Danio rerio	FoxA3_drer	NP_571374	NM_131299.1
Gallus gallus	FoxA2_ggal	NP_990101	NM_204770.1
Homo sapiens	FOXA1_hsap	NP_004487	NM_004496.2
Homo sapiens	FOXA2_hsap	NP_068556	NM_021784.3
Homo sapiens	FOXA3_hsap	NP_004488	NM_004497.2

Mus musculus	Foxa1_mmus	NP_032285	NM_008259.1
Mus musculus	Foxa2_mmus	NP_034576	NM_010446.1
Mus musculus	Foxa3_mmus	NP_032286	NM_008260.1
Oreochromis mossambicus	HNF3B_omos [Φ]	AAL68498	AF251499.1
Oryzias latipes	FoxA2_olat [Φ]	O42097	AB001572
Oryzias latipes	FoxA3_olat [Φ]	BAA23580	AB001573.2
Patella vulgata	fkh_pvul	CAD45552	AJ507424.1
Rattus norvegicus	FoxA1_rnor	NP_036874	NM_012742.1
Rattus norvegicus	FoxA2_rnor	NP_036875	NM_012743.1
Rattus norvegicus	FoxA3_rnor	NP_058773	NM_017077.1
Tetraodon nigroviridis	UN_3_tnig [Φ]	CAF89623	CAAE01007089
Tetraodon nigroviridis	UN_45_tnig [Φ]	CAG09884	CAAE01015009
Tetraodon nigroviridis	UN_51_tnig [Φ]	CAG12727	CAAE01015113
Tribolium castaneum	Tcfkh_tcas	AAF71998	AF217810.1
Xenopus laevis	FoxA1b_xlae	P32315	M93658
Xenopus laevis	FoxA4a_xlae	P33205	X65171
Xenopus laevis	FoxA4b_xlae	P33206	S93559
Xenopus tropicalis	FoxA1_xtro [Φ]	NP_989419	NM_204088.1
Xenopus tropicalis	FoxA2_xtro [Φ]	NP_989423	NM_204092.1

FoxD Cluster 24 sequences

Species	Sequence Identifier	Protein Accession #	Nucleotide Accession #
Danio rerio	FoxD3_drer	NP_571365	NM_131290.1
Danio rerio	FoxD5_drer	NP_571345	NM_131270.1

Gallus gallus	FoxD1_ggal	NP_990523	NM_205192.1
Gallus gallus	FoxD2_ggal	NP_990283	NM_204952.1
Gallus gallus	FoxD3_ggal	NP_990282	NM_204951.1
Gorilla gorilla	FoxD4_ggor* [Φ]	AAQ72340	AY345862.1
Homo sapiens	FOXD1_hsap	NP_004463	NM_004472.1
Homo sapiens	FOXD2_hsap	NP_004465	NM_004474.2
Homo sapiens	FOXD3_hsap	NP_036315	NM_012183.1
Homo sapiens	FOXD4b_hsap* [Φ]	NP_954714	NM_199244.1
Homo sapiens	FOXD4L2_hsap* [Φ]	NP_954586	NM_199135.1
Homo sapiens	FOXD4L3_hsap* [Φ]	NP_955390	NM_199358.1
Mus musculus	Foxd1_mmus	NP_032268	NM_008242.1
Mus musculus	Foxd2_mmus	NP_032619	NM_008593.1
Mus musculus	Foxd3_mmus	NP_034555	NM_010425.2
Oreochromis mossambicus	FoxD5_omos [Φ]	AAM75747	AF251498.1
Pan troglodytes	FoxD4_ptro* [Φ]	NP_001009014	NM_001009014
Tetraodon nigroviridis	UN_48_tnig [Φ]	CAG11584	CAAE01015039
Xenopus laevis	FoxD2_xlae	CAC69867	AJ344435.1
Xenopus laevis	FoxD3b_xlae	CAC12895	AJ298866.1
Xenopus laevis	xfd12_xlae [Φ]	CAB44728	AJ242676.1
Xenopus laevis	xfd12dblprime_xlae [Φ]	CAB44730	AJ242678.1
Xenopus laevis	xfd12prime_xlae	CAB44729	AJ242677.1
Xenopus laevis	xfd6_xlae [Φ]	BAA36334	AB014611.1

FoxI Cluster 10 sequences

**Species**	**Sequence Identifier**	**Protein Accession #**	**Nucleotide Accession #**

Danio rerio	FoxI1_drer	NP_859424	NM_181735.1
Danio rerio	FoxI2_drer	NP_944598	NM_198916.1
Danio rerio	FoxI3a_drer	NP_944599	NM_198917.1
Danio rerio	FoxI3b_drer	NP_944600	NM_198918.1
Homo sapiens	FOXI1_hsap	NP_036320	NM_012188.3
Mus musculus	Foxi1_mmus	NP_076396	NM_023907.2
Mus musculus	Foxi2_mmus	NP_899016	NM_183193.1
Tetraodon nigroviridis	UN_46_tnig	CAG10122	CAAE01015015
Xenopus laevis	FoxI1_xlae	AAH42303	BC042303.1
Xenopus laevis	FoxI1c_xlae	CAD31849	AJ487620.1

FoxO Cluster 12 sequences

Species	Sequence Identifier	Protein Accession #	Nucleotide Accession #
Danio rerio	FoxO5_drer	NP_571160	NM_131085.1
Homo sapiens	FOXO1a_hsap	NP_002006	NM_002015.2
Homo sapiens	FOXO3a_hsap	NP_001446	NM_001455.2
Homo sapiens	FOXO4_hsap	NP_005929	NM_005938.1
Mus musculus	Foxo1_mmus	NP_062713	NM_019739.2
Mus musculus	Foxo3_mmus	NP_062714	NM_019740.1
Mus musculus	Foxo4_mmus	NP_061259	NM_018789.1
Mus musculus	Foxo6_mmus	NP_918949	NM_194060.1
Spermophilus tridecemlineatus	FoxO1a_stri	AAO72710	AY255525.1
Sus scrofa	FoxO1a_sscr	NP_999179	NM_214014.1
Tetraodon nigroviridis	UN_53_tnig*	CAG13202	CAAE01015123

Xiphophorus maculatus	FoxO5_xmac $^{\Phi}$	AAK74186	AY040320.1

FoxP Cluster 10 sequences

**Species**	**Sequence Identifier**	**Protein Accession #**	**Nucleotide Accession #**
Gorilla gorilla	FoxP2_ggor $^{\Phi}$	AAN03386	AF512948.1
Homo sapiens	FOXP1_hsap $^{\Phi}$	NP_116071	NM_032682.4
Homo sapiens	FOXP2_hsap $^{\Phi}$	NP_055306	NM_014491.1
Homo sapiens	FOXP4_hsap	NP_612466	NM_138457.2
Macaca mulatta	FoxP2_mmul $^{\Phi}$	AAN03388	AF512950.1
Mus musculus	Foxp1_mmus $^{\Phi}$	NP_444432	NM_053202.1
Mus musculus	Foxp2_mmus $^{\Phi}$	NP_444472	NM_053242.3
Pan troglodytes	FoxP2_ptro $^{\Phi}$	Q8MJA0	AY143178
Pongo pygmaeus	FoxP2_ppyg $^{\Phi}$	AAN03387	AF512949.1
Taeniopygia guttata	FoxP2_tgut $^{\Phi}$	AAR28756	AY395709.1
