# Supplementary material for: Identification and analysis of evolutionary selection pressures acting at the molecular level in five forkhead subfamilies
Source: BMC Evol Biol. 2008 Sep 24;8:261. doi: 10.1186/1471-2148-8-261 (PMC2570691; doi:10.1186/1471-2148-8-261)
Supplement: Additional file 2 — Alignment procedure with ClustalX and ClustalW. The procedure used to create multiple sequence alignments is provided in this file. [file 1471-2148-8-261-S2.pdf]

Additional file 2

Alignment Procedure with ClustalX and ClustalW

The first step of the alignment procedure was optimization of the substitution matrices, BLOSUM, Gonnet or PAM, used during the pairwise and multiple alignment phases. A full multiple alignment was performed with CLUSTALX using each combination of pairwise and multiple alignment substitution matrices with all other parameters at default values. A neighbor-joining tree was constructed from each alignment using the CLUSTALX program. The quality scores, using default parameters, assigned by CLUSTALX to each column in the alignment were averaged over all sites by the program Tune ClustalX [1], giving an overall Q-score for the alignment. The neighbor-joining trees were then examined to determine the most common topology. The alignment producing a neighbor-joining tree with the common topology and the highest Q-score was considered to be made with the optimal combination of pairwise and multiple alignment substitution matrices. In all cases, the alignment that produced the highest Q-score also produced a neighbor-joining tree with the most common topology. The next step in aligning the sequences was optimization of the gap open and gap extension penalties used during the multiple alignment phase. The dendrogram produced by the alignment that optimized the substitution matrices was used during this step so that the pairwise gap penalty parameters did not need to be optimized. The optimal substitution matrix for the data set, determined during the first step of the alignment, was also used during this phase. Alignments were created with CLUSTALW varying the gap open penalty from five to fifteen in steps of one and the gap extension penalty from zero to three in steps of 0.5 also including the default value of 0.2. These ranges were chosen based on previous studies [2-5]. CLUSTALW was used instead of CLUSTALX so that the alignments could be automated through use of a Perl script. Q-scores were then calculated for each alignment and the alignment with the highest Q-score was considered to be made with the optimal combination of gap penalties. This alignment was used for all further analyses.

## Literature Cited


1. Hall BG: **Improving ClustalX alignments with TuneClustalX.** In.: Bellingham Research Institute; 2004.
2. Gotoh O: **Significant improvement in accuracy of multiple protein sequence alignments by iterative refinement as assessed by reference to structural alignments.** *Journal of Molecular Biology* 1996, **264**:823-838.
3. Higgins DG, Thompson JD, Gibson TJ: **Using CLUSTAL for multiple sequence alignments.** *Methods Enzymol* 1996, **266**:383-402.
4. Vogt G, Etzold T, Argos P: **An assessment of amino acid exchange matrices in aligning protein sequences: the twilight zone revisited.** *J Mol Biol* 1995, **249**:816-831.
5. Yuan J, Amend A, Borkowski J, DeMarco R, Bailey W, Liu Y, Xie G, Blevins R: **MULTICLUSTAL: a systematic method for surveying Clustal W alignment parameters.** *Bioinformatics* 1999, **15**(10):862-863.
