# Supplementary material for: Identification and analysis of evolutionary selection pressures acting at the molecular level in five forkhead subfamilies
Source: BMC Evol Biol. 2008 Sep 24;8:261. doi: 10.1186/1471-2148-8-261 (PMC2570691; doi:10.1186/1471-2148-8-261)
Supplement: Additional file 3 — Amino acid alignments. The amino acid alignment of each of the clusters analyzed (A. FoxA, B. FoxD, C. FoxI, D. FoxO and E. FoxP) with regions of interest highlighted is shown here. [file 1471-2148-8-261-S3.pdf]

Additional file 3

Amino Acid Alignments

A. Alignment of the FoxA cluster of sequences.

Conserved and functionally important regions noted in the literature are highlighted. Peach: conserved domain IV [1, 2] Blue: conserved domain V [1, 2] Black Box: nuclear localization signal [2] Green: forkhead domain (NCBI Protein database, see Additional file 1 for accession numbers) Yellow: conserved domain II [3] Red Box: EH1 motif [4, 5] Pink: conserved domain III [3] Positively selected sites identified by branch-site analysis are indicated by purple boxes.

```
FoxA4a_xlae      -MLNRVKLEIKDPMDWN--TMYQENEMYSGIHN-------------MTNVLPSNSFLPND 44
FoxA4b_xlae      -MLNRVKLELKDPMDWN--TMYQENEIYSGIHN-------------MTNGLPSNSFLPTD 44
FoxA4_amex       -MLNGIKLENQEAMDWT--HFYQDNEVYSGVHS-------------MPSSLAS-TYIPND 43
FoxA3_olat       -MLSSVKMEAHDITDWNTTFYSEASEMYSSP---SATG--------LGSMGSINSYINLN 48
UN_51_tnig       -MLSSVKMETHDLPEWN-TFYSEASEMYSSP---STMNSGLAS---MGSMGSINSYINLN 52
FoxA3_drer       -MLSSVKMESHEIPEWN-PFYSEANEMYSSP---SAMN----------SVSSLNSYINLN 45
Foxa3_mmus       -MLGSVKMEAHDLAEWS--YYPEAGEVYSPVN-------------PVPTMAPLNSYMTLN 44
FoxA3_rnor       -MLGSVKMEAHDLAEWS--YYPEAGEVYSPVN-------------PVPTMAPLNSYMSLN 44
FOXA3_hsap       -MLGSVKMEAHDLAEWS--YYPEAGEVYSPVT-------------PVPTMAPLNSYMTLN 44
FoxA2_clal       MMLGAVKMEGHEHT-DWSTYYGEPECYTSVGNMNTGLG---MNSMNTYMSMSGMST---G 53
HNF3b_omos       MMLGAVKMEGHEHT-DWSTYYGEPECYTSVGNMNAGLG---MNSMNTYMSMSGMST---T 53
UN_45_tnig       -MLGAVKMEGHEHT-DWSTYYAEPECYTSVGNMNTGLG---MNSMNTYMSMSGMNT---T 52
FoxA2_olat       MMLGAVKMEGHEHT-DWSTYYGEPECYTSVGNMNTGLG---MNSMNTYMSMSGMST---T 53
FoxA2_drer       -MLGAVKMEGHEHAADWSTYYGEPECYTSVSNMNTGLG---MNSMNTYMTMSGMSS---T 53
FoxA2_xtro       -MLGAVKMEGHEAT-DWSSYYGEPEAYSSVGNMNAGLS---MNPMNTYMSMSAMST---S 52
Foxa2_mmus       -MLGAVKMEGLEPS-DWSSYYAEPEGYSSVSNMNAGLG---MNGMNTYMSMSAAAMGGGS 55
FoxA2_rnor       -MLGAVKMEGHEPS-DWSSYYAEPEGYSSVSNMNASLG---MNGMNTYMSMSAAAMGSGS 55
FOXA2_hsap       -MLGAVKMEGHEPS-DWSSYYAEPEGYSSVSNMNAGLG---MNGMNTYMSMSAAAMGSGS 55
FoxA2_ggal       -MLGAVKMEGHEHT-DWSNYYGEPESYSSVSNMNAGLG---MNSMNTYMTMSAMST---T 52
Foxa1_mmus       -MLGTVKMEGHESNDWNSYYADTQEAYSSVPVSNMNSG---LGSMNSMNTYMTMNTMTTS 56
FoxA1_rnor       -MLGTVKMEGHESNDWNSYYADTQEAYSSVPVSNMNSG---LGSMNSMNTYMTMNTMTTS 56
FOXA1_hsap       -MLGTVKMEGHETSDWNSYYADTQEAYSSVPVSNMNSG---LGSMNSMNTYMTMNTMTTS 56
FoxA1b_xlae      -MLGIVKMEGHETTDWSNYYQDAQEGYSSVPVSNMPQG---LATMN---TYMTMNPMSSG 53
FoxA1_xtro       -MLGIVKMEGHETTDWSNYYQDTQEAYSSVPVSNMTQG---LASMN---TYMTMNPMSSS 53
UN_3_tnig        -----------------------------MAATGLGSG---LGSMTG---------YMSS 19
AmHNF31_bflo     -------------------MLSAPKGYPTG----------------TTMNTMGMNTMSSM 25
HNF3_bflo        ---MLSPKSAYEAQGGSPSSMQAMTAMTTGNSY-----------SPSSYTSSGYSVTQSM 46
SGF1_bmor        --MISQKLSYGDVPTS--------ASLSSLSPGLAPPY---VNGMGCMPAQPYPN----- 42
Tcfkh_tcas       --MLTQKLYSDSTTMA--------TSSNAMSPMTPTYS---MNSMSCVSMPSMNCSPQGA 47
At.fkh_atep      --MLTHKSFPDCSTMP---------SGNYMPVTSMPMS---LNYSTPQFNAGLLSP---- 42
fkh_pvul         --MLSAKPGSYDPTSSGGYSMASMTSINTMGGVGPMSN---MNYPSQGMGGMHGAMSSMN 55


FoxA4a_xlae      VSTVTTS---MPYMSNGLPGPV------------TSIQGNIGSLGSMPQGMVGSLAPP-- 87
FoxA4b_xlae      VPTVTSS---MTYMSNGLPGPV------------ASIQGNLGSLGSMTQGMVGSLAPP-- 87
FoxA4_amex       IHVASAG---MNYMNPGLGCSV------------PALPGGGSPVNSMAPGSLNIASPLNQ 88
FoxA3_olat       AAAS-PTAMNMPYPSSSLSSS------------LAPMGSGPNHMSLSPVASSLSSGSLT 95
UN_51_tnig       AATASPASMNMAYPSSSLSSST------------LASMGSGPTHMSLSPVASSLASGPLT 100
FoxA3_drer       SACS-TSSMNMGYPSAGLNSSP------------LSSMGGGPNHMSLSPVGSSLNPSSLT 92
Foxa3_mmus       PLSS-------PYPPGGLQASP------------LPTG-------PLAPPAPTAPLGPTF 78
FoxA3_rnor       PLSS-------PYPPGGLQASP------------LPTG-------PLAPPAPTAPLGPTF 78
FOXA3_hsap       PLSS-------PYPPGGLPASP------------LPSG-------PLAPPAPAAPLGPTF 78
FoxA2_clal       ANMTAN-SMNMSYVNTGMSPSMTGMSPG------TGAMNGMG-----AGMTAMGAALSPS 101
HNF3b_omos       ANMTAN-SMNMSYVNTGMSPSMTGMSPG------TGAMNGMG-----AGMTAMSAALSPS 101
UN_45_tnig       ANMTAN-SMNMSYVNTGMSPSMTGMSPG------TGAMNGMG-----AGMTAMSAALSPS 100
FoxA2_olat       ANMTAN-SMNMSYVNTGMSPSMTGMSPG------TGAMNGMG-----AGMTAMSTALSPS 101
FoxA2_drer       ANMTAANTMNMSYVNTGMSPSMTGMSPG------TGAMAGMG-----AGMTGMSAALSPT 102
FoxA2_xtro       ANMTAG-SMNMSYVNTGMSPSLTGMSPG------TGAMTGMG-----TGVASMASHLSPS 100
Foxa2_mmus       GNMSAGSMNMSSYVGAGMSPSLAGMSPG------AGAMAGMSGSAGAAGVAGMGPHLSPS 109
FoxA2_rnor       GNMSAGSMNMSSYVGAGMSPSLAGMSPG------AGAMAGMSGSAGAAGVAGMGPHLSPS 109
FOXA2_hsap       GNMSAGSMNMSSYVGAGMSPSLAGMSPG------AGAMAGMGGSAGAAGVAGMGPHLSPS 109
FoxA2_ggal       ANMTAATSMNMSYANTGMSPSLAGMSPG------AGAMAGMG----SAGVAGMGAHLSPS 102
Foxa1_mmus       GNMTPA-SFNMSYANTGLGAGLSPGAVAGMPGASAGAMNSMTAAGVTAMGTALSPGGMGS 115
FoxA1_rnor       GNMTPA-SFNMSYANPGLGAGLSPGAVAGMPGGSAGAMNSMTAAGVTAMGAALSPGGMGS 115
FOXA1_hsap       GNMTPA-SFNMSYANPGLGAGLSPGAVAGMPGGSAGAMNSMTAAGVTAMGTALSPSGMGA 115
FoxA1b_xlae      SNITSG-SFNMPYGNSGLGAGLSPSGMSGMGS--AGAMNGMGSG-VPSMGSALSPSNMNA 109
FoxA1_xtro       SNMTAG-SFNMSYANSGLGAGLSPSGMSGMGAGSASAMNGMGSG-VSSMGTALSPSSMNA 111
UN_3_tnig        GGTTAG-SFNMSYS----GSALSPPPVAGMSSSTPAAMSGLGGG-MAPMGGPLSPSHMNS 73
AmHNF31_bflo     GGMNHASYTGTGPVNPGAYSAS------------AYSGTMNGMGTG-------------- 59
HNF3_bflo        TTLGSSYSTGMNGVGMGTIPPS------------HTSMTTMGMNSVPGSQLTTMSTMGAQ 94
SGF1_bmor        ---------LYSNNMVAGGSCMGSPSVGYSPP----------------STMASCMGGAGA 77
Tcfkh_tcas       SFGSSMLNSGMPGGMAMNGNGMTSSSMGYTTIGSP---ISNRIRHEMATPMATMNSYGSV 104
At.fkh_atep      ------------QNMAAVAPACMTQPMP------------------PIGSITPLNNVAPN 72
fkh_pvul         TMPPSMGSMGMGGMAAMHGSSMHGSMTAMNPMNTMGSMNSMGAMGSMNGMSSIGSMSSMN 115
```

```
FoxA4a_xlae      -------------PSTAAYPLGYCQGESE-----------FQRDPRTYRRNYSHAKPPYS 123
FoxA4b_xlae      -------------PSTSAYPLGYCQGESE-----------FQRDPRTYRRNYSHAKPPYS 123
FoxA4_amex       SMNVAPQGSSSMGSYTPMNTLAYGQGGLD-----------YQRDPRAYRRNYSHAKPPYS 137
FoxA3_olat       QLGPAAAGSLGPLSHYQNMGQSMSQLGYPSTGSLSRSSP-KEIPPKPYRRSLTHAKPPYS 154
UN_51_tnig       QLTTAPPASLGSLPHYQNMGQSMGQLGYTSNASLTRTGP-KEIPPKPYRRSLTHAKPPYS 159
FoxA3_drer       QLG-SSASTLGPLSHYQSMGQPMSQISYPSPTSLNR----TKEMPKPYRRSLTHAKPPYS 147
Foxa3_mmus       PSLGTGGSTGGSASGYVAPGPGLVHG---------------KEMAKGYRRPLAHAKPPYS 123
FoxA3_rnor       PGLGAGSGTGGSASGYGAPGPGLVHG---------------KEMAKGYRRPLTHAKPPYS 123
FOXA3_hsap       PGLGVSGGS--SSSGYGAPGPGLVHG---------------KEMPKGYRRPLAHAKPPYS 121
FoxA2_clal       MSPMTAQPA-SMNALTSYSNMNAMSPIYGQSNINRSRD------PKTYRRSYTHAKPPYS 154
HNF3b_omos       MSPMTAQPA-SMNALTSYSNMNAMSPIYGQSNINRSRD------PKTYRRSYTHAKPPYS 154
UN_45_tnig       MSPMTAQPA-SMNALTSYTNMNAMSPIYGQSNINRSRD------PKTYRRSYTHAKPPYS 153
FoxA2_olat       MSPMTGQPG-SMNALTSYTNMNAMSPIYGQSNINRSRD------PKTYRRSYTHAKPPYS 154
FoxA2_drer       MSPMAAQAP-SMNALTSYSNMNAMSPMYGQSNINRSRD------PKTYRRSYTHAKPPYS 155
FoxA2_xtro       MSPMSAQAT-SMNALAPYTNMNSMSPIYGQSNINRSRD------PKTYRRSYTHAKPPYS 153
Foxa2_mmus       LSPLGGQAAGAMGGLAPYANMNSMSPMYGQAGLSRARD------PKTYRRSYTHAKPPYS 163
FoxA2_rnor       LSPLGGQAAGAMGGLAPYANMNSMSPMYGQAGLSRARD------PKTYRRSYTHAKPPYS 163
FOXA2_hsap       LSPLGGQAAGAMGGLAPYANMNSMSPMYGQAGLSRARD------PKTYRRSYTHAKPPYS 163
FoxA2_ggal       MSPMGGQAG-SMNALAPYTNMNSMSPIYGQSNLNRSRD------PKTYRRSYTHAKPPYS 155
Foxa1_mmus       MGAQPATSMNGLGPYAAAMNPCMSPMAYAPSNLGRSRAG-GGGDAKTFKRSYPHAKPPYS 174
FoxA1_rnor       MGAQPAASMNGLGPYAAAMNPCMSPMAYAPSNLGRSRAG-GGGDAKTFKRSYPHAKPPYS 174
FOXA1_hsap       MGAQQAASMNGLGPYAAAMNPCMSPMAYAPSNLGRSRAG-GGGDAKTFKRSYPHAKPPYS 174
FoxA1b_xlae      IQSAQQASMNSLS--YSSMNSGMSPMGYGATNINRTRD------SKTFRRSYPHAKPPYS 161
FoxA1_xtro       MS-AQQASINSLS--YSGMNPGMSPMAYGPSNMNRTRD------TKTFRRSYPHAKPPYS 162
UN_3_tnig        VPAQQGSLG---LSPYGGMSPSSSMAYSGGGGMNRARD------NKAFRRSYPHAKPPYS 124
AmHNF31_bflo     -YPAAGGMGGLQSYPAGSVNAMGTMQTMN--NMALNRNA-IAEREKAYRRSYTHAKPPYS 115
HNF3_bflo        HPGLANSLGVGMAHPGQSMSPMSMQGSVNGVNMNLTRED-VLNRQKQYRRSYTHAKPPYS 153
SGF1_bmor        VPYGSLPREQEAASPTSALQRAR--------------------NDKTYRRSYTHAKPPYS 117
Tcfkh_tcas       GTLGRGDLGGGDTSPNSALQRAR--------------------ADKTYRRSYTHAKPPYS 144
At.fkh_atep      RTDMQTYVNTDYHSDPNLALKAR--------------------NDK-FRRSLPHAKPPYS 111
fkh_pvul         GMNRQMDPNMMSMDRAQALNRA---------------------RDKNYRRSYTHAKPPYS 154

FoxA4a_xlae      YISLITMAIQQAPNKMMTLNEIYQWIIDLFPYYRQNQQRWQNSIRHSLSFNDCFVKVPRS 183
FoxA4b_xlae      YISLITMAIQQAPNKMMTLNEIYQWIVDLFPYYRQNQQRWQNSIRHSLSFNDCFIKVPRS 183
FoxA4_amex       YISLITMAVQQSPNKMMTLNEIYQWITDLFPYYRQNQQSWQNSIRHSLSFNDCFVKVPRS 197
FoxA3_olat       YISLITMAIQQSGSKMLTLNEIYQWIMDLFPYYRENQQRWQNSIRHSLSFNDCFVKVARS 214
UN_51_tnig       YISLITMAIQQSSSKMLTLNEIYQWIMDLFPYYRENQQRWQNSIRHSLSFNDCFVKVARS 219
FoxA3_drer       YISLITMAIQQSQSKMLTLNEIYQWIMDLFPYYRENQQRWQNSIRHSLSFNDCFVKVARS 207
Foxa3_mmus       YISLITMAIQQAPGKMLTLSEIYQWIMDLFPYYRENQQRWQNSIRHSLSFNDCFVKVARS 183
FoxA3_rnor       YISLITMAIQQAPGKMLTLSEIYQWIMDLFPYYRENQQRWQNSIRHSLSFNDCFVKVARS 183
FOXA3_hsap       YISLITMAIQQAPGKMLTLSEIYQWIMDLFPYYRENQQRWQNSIRHSLSFNDCFVKVARS 181
FoxA2_clal       YISLITMAIQQSPSKMLTLAEIYQWIMDLFPFYRQNQQRWQNSIRHSLSFNDCFVKVPRS 214
HNF3b_omos       YISLITMAIQQSPSKMLTLAEIYQWIMDLFPFYRQNQQRWQNSIRHSLSFNDCFVKVPRS 214
UN_45_tnig       YISLITMAIQQSPSKMLTLAEIYQWIMDLFPFYRQNQQRWQNSIRHSLSFNDCFVKVPRS 213
FoxA2_olat       YISLITMAIQQSPSKMLTLAEIYQWIMDLFPFYRQNQQRWQNSIRHSLSFNDCFLKVPRS 214
FoxA2_drer       YISLITMAIQQSPSKMLTLSEIYQWIMDLFPFYRQNQQRWQNSIRHSLSFNDCFLKVPRS 215
FoxA2_xtro       YISLITMAIQQSPNKMLTLSEIYQWIMDLFPFYRQNQQRWQNSIRHSLSFNDCFLKVPRS 213
Foxa2_mmus       YISLITMAIQQSPNKMLTLSEIYQWIMDLFPFYRQNQQRWQNSIRHSLSFNDCFLKVPRS 223
FoxA2_rnor       YISLITMAIQQSPNKMLTLSEIYQWIMDLFPFYRQNQQRWQNSIRHSLSFN-DFLKVPRA 222
FOXA2_hsap       YISLITMAIQQSPNKMLTLSEIYQWIMDLFPFYRQNQQRWQNSIRHSLSFNDCFLKVPRS 223
FoxA2_ggal       YISLITMAIQQSPNKMLTLSEIYQWIMDLFPFYRQNQQRWQNSIRHSLSFNDCFLKVPRS 215
Foxa1_mmus       YISLITMAIQQAPSKMLTLSEIYQWIMDLFPYYRQNQQRWQNSIRHSLSFNDCFVKVARS 234
FoxA1_rnor       YISLITMAIQQAPSKMLTLSEIYQWIMDLFPYYRQNQQRWQNSIRHSLSFNACFVKVARS 234
FOXA1_hsap       YISLITMAIQQAPSKMLTLSEIYQWIMDLFPYYRQNQQRWQNSIRHSLSFNDCFVKVARS 234
FoxA1b_xlae      YISLITMAIQQAPSKMLTLSEIYQWIMDLFPYYRQNQQRWQNSIRHSLSFNDCFIKVSRS 221
FoxA1_xtro       YISLITMAIQQAPSKMLTLSEIYQWIMDLFLYYRQNQQRWQNSIRHSLSFNDCFVKVARS 222
UN_3_tnig        YISLITMAIQQAPSKMLTLSEIYQWIMDLFPYYRQNQQRWQNSIRHSLSFNDCFVKVSRS 184
AmHNF31_bflo     YISLITMSIQSSPNKMVTLAEIYQFIMDLFPYYRQNQQRWQNSIRHSLSFNDCFVKVPRT 175
HNF3_bflo        YIALITMAVQSSPNKMVTLSEIYQFIMDLFPFYRQNQQRWQNSIRHSLSFNDCFVKVQRT 213
SGF1_bmor        YISLITMAIQNNPSRMLTLSEIYQFIMDLFPFYRQNQQRWQNSIRHSLSFNDCFVKVPRT 177
Tcfkh_tcas       YISLITMAIQNSPQKMLTLSEIYQFIMDLFPFYRQNQQRWQNSIRHSLSFNDCFVKVPRT 204
At.fkh_atep      YISLITMAIQNSPQKMLTLNEIYQFIVDIFPFYRQNQQRWQNSIRHSLSFNDCFVKVART 171
fkh_pvul         YISLITMAIQQSPNKMCTLSEIYQFIMDLFPFYRQNQQRWQNSIRHSLSFNDCFVKVPRT 214
```

```
FoxA4a_xlae     PEKPGKGSYWTLHPESGNMFENGCYLRRQKRFKCER---SKSG---------------EG 225
FoxA4b_xlae     PEKPGKGSYWTLHPESGNMFENGCYLRRQKRFKCER---SKSG---------------EG 225
FoxA4_amex      PEKPGKGSYWALHPDSGNMFENGCYLRRQKRFKCDRRSGSKRV---------------QD 242
FoxA3_olat      PDKPGKGSYWTLHPQSGNMFENGCYLRRQKRFKIEDKASKKG----------------SK 258
UN_51_tnig      PDKPGKGSYWTLHPQSGNMFENGCYLRRQKRFKIEDKAKKGK----------------SQ 263
FoxA3_drer      PDKPGKGSYWALHPNSGNMFENGCYLRRQKRFKIEEKAGKKSS---------------SK 252
Foxa3_mmus      PDKPGKGSYWALHPSSGNMFENGCYLRRQKRFKLEEKAKKGN------------------ 225
FoxA3_rnor      PDKPGKGSYWALHPSSGNMFENGCYLRRQKRFKLEEKAKKGN------------------ 225
FOXA3_hsap      PDKPGKGSYWALHPSSGNMFENGCYLRRQKRFKLEEKVKKGG------------------ 223
FoxA2_clal      PDKPGKGSFWTLHPDSGNMFENGCYLRRQKRFKCDKKMGMKDG----------------G 258
HNF3b_omos      PDKPGKGSFWTLHPDSGNMFENGCYLRRQKRFKCDKKMKEP------------------G 256
UN_45_tnig      PDKPGKGSFWTLHPDSGNMFENGCYCG--------------------------------A 241
FoxA2_olat      PDKPGKGSFWTLHPDSGNMFENGCYLRRQKRFKCEKKMSMKEP----------------G 258
FoxA2_drer      PDKPGKGSFWTLHPDSGNMFENGCYLRRQKRFKCDKKLSKD-----------------PS 258
FoxA2_xtro      PDKPGKGSFWTLHPDSGNMFENGCYLRRQKRFKCEKKPSLREG---------------GG 258
Foxa2_mmus      PDKPGKGSFWTLHPDSGNMFENGCYLRRQKRFKCEKQLALKEA----------------A 267
FoxA2_rnor      PDKPGKGSFWTLHPDSGNMFENGCYLRRQKRFKCENELALKEA----------------A 266
FOXA2_hsap      PDKPGKGSFWTLHPDSGNMFENGCYLRRQKRFKCEKQLALKEA----------------A 267
FoxA2_ggal      PDKPGKGSFWTLHPDSGNMFENGCYLRRQKRFKCEKQLATKDG----------------G 259
Foxa1_mmus      PDKPGKGSYWTLHPDSGNMFENGCYLRRQKRFKCEKQPGAGGG--------SGGGGSKGG 286
FoxA1_rnor      PDKPGKGSYWTLHPDSGNMFENGCYLRRQKRFKCEKQPGAGGG--------SGGGGSKGV 286
FOXA1_hsap      PDKPGKGSYWTLHPDSGNMFENGCYLRRQKRFKCEKQPGAGGGGGSG----SGGSGAKGG 290
FoxA1b_xlae     PDKPGKGSYWTLHPDSGNMFENGCYLRRQKRFKCEKTQ-----------------GGKGN 264
FoxA1_xtro      PDKPGKGSYWTLHPDSGNMFENGCYLRRQKRFKCEKQQ-----------------GGKGS 265
UN_3_tnig       PDKPGKGSYWTLHPDSGNMFENGCYLRRQKRFKCEKKTSLKCDG---------------- 228
AmHNF31_bflo    PDRPGKGSYWTLHPEAGNMFENGCYLRRQKRFKCEKKLAMKMAQQQA----------APT 225
HNF3_bflo       PDRPGKGSYWTLHPNAHSMFENGCYLRRQKRFKCERKAALKAEQKAE----------NEE 263
SGF1_bmor       PDKPGKGSFWTLHPDSGNMFENGCFLRRQKRFKDEKKETLRQA----------------- 220
Tcfkh_tcas      PDKPGKGSFWSLHPDSGNMFENGCYLRRQKRFKDEKKELIRQT----------------- 247
At.fkh_atep     PDKPGKGSFWALHPESGDMFENGCFLRRQKRFKCTKKEAIRQT----------------- 214
fkh_pvul        PDRPGKGSYWALHPDSGNMFENGCYLRRQKRFKCLKKESMRSS----------------- 257

FoxA4a_xlae     E-KKVNKPGEETGGNLKENPLGYDDCSSSRSPQAAVNDGGRDSTGSSIHQACGSSPVGLS 284
FoxA4b_xlae     E-RKGNKPGDETGGSLKETPVSFDDCSSSRSPQAAVNDGGRDSTGSSIHQATGGSPVGFS 284
FoxA4_amex      E-NGSHQPCEAAIKPLILHLRAPTEFSNPFTRASAGLEHNHAMMSQQQPNMAASSPSISP 301
FoxA3_olat      NQEGGSGKGSHSGDHLVENHSPAGGSEGPDSAHSDNSHPGSSDDQPHQRNSLVPLDCPQL 318
UN_51_tnig      E---GSGKGNHSGDHLAEDHSPTGGSEGADSAHSDNSHPGSSDDQQHSRNSLVPLDCPPL 320
FoxA3_drer      SQDGSSTKGTHSSEGMQEEHSPTTGSDGAESAHSDSSHAGSTSEE--QQRSLVQLDCPSQ 310
Foxa3_mmus      -----SATSASRNGTAGSATSATTTAATAVTSPAQPQPTP-SEPEAQSGDDVGGLDCASP 279
FoxA3_rnor      -----SATSATRNGTVGSATSATTTAATAVTSPAQPQPTPPSEPEAQSGEDVGGLDCASP 280
FOXA3_hsap      -----SGAATTTRNGTGSAASTTTPAATVTSPPQPPPPAP--EPEAQGGEDVGALDCGSP 276
FoxA2_clal      RKSGDGGSS-----------------NSSSESCNGNESPHSNSS-SGEHKRSLSDMKSSQA 301
HNF3b_omos      RKSGDGGSS-----------------NSSSESCNGNESPLSNSS-SSDHKRSLSDMKTSQA 299
UN_45_tnig      RSGSNGGSS-----------------NSSSESCNGNESPHSNSS-SSEHKRSLSDMKTSQA 284
FoxA2_olat      RKGGDGGSA-----------------NSSSDSCNGNESPHSNSS-SGEHKRSLSDMKGSQA 301
FoxA2_drer      RKTSEGGS------------------NSSSESCNGNESPHSNSS-SNELKRSLSDMKSGQG 300
FoxA2_xtro      KKLSEGSSSVGS-----------AANSSSESSVGNESPHSSSSPCQEQKRSLVDMKSSQG 307
Foxa2_mmus      GAASSGGKKTAPGSQASQAQLGEAAGSASETPAGTESPHSSASPCQEHKRGGLSELKGAP 327
FoxA2_rnor      GAGSGGGKKTAPGTQASQVQLGEAAGSASETPAGTESPHSSASPCQEHKRGGLSELKGTP 326
FOXA2_hsap      GAAGSG-KKAAAGAQASQAQLGEAAGPASETPAGTESPHSSASPCQEHKRGGLGELKGTP 326
FoxA2_ggal      GG------KKGPGQPPSQPLGEGSSSGGSEGSAGAESPAS-ASPCRDNKR-ALAELKGAA 311
Foxa1_mmus      PESRKDPSGPGNPSAESPLHRGVHGKASQLEGAPAPG-PAASPQTLDHSGATATGGASEL 345
FoxA1_rnor      PENRKDPSGPVNPSAESPIHRGVHGKASQLEGAPAPG-PAASPQTLDHSGATATGGGSEL 345
FOXA1_hsap      PESRKDPSGASNPSADSPLHRGVHGKTGQLEGAPAPG-PAASPQTLDHSGATATGGASEL 349
FoxA1b_xlae     QDGRKDHSGPS-----SPLHR-VHGKSSQMDSSSSMSNPSSSPQALEHNGSNGEMKPQVA 318
FoxA1_xtro      QDGRKDVSGPS-----SPLHR-VHGKSSQMDSSSSMSNPSSSPQSLEHNGSNGEMKPQVA 319
UN_3_tnig       ---------------RKEEGGGASPSGDKPGGLLVSSSQAASPPGLDLQGGTPDLKVSSS 273
AmHNF31_bflo    PPTPGRELRRLADYHGSTPTTTSNGASTLQPLQPINTPSPNPQEQQQHQHHQHQHQQQHQ 285
HNF3_bflo       VLSGAPAGQQPPPQANTPVHNSPTPESTHVSSSPMTVTTQTPSP----------TLTQLT 313
SGF1_bmor       ----QKAQQTHGHHGGSHDKRGEHGHDKSAPPGPGEDKEMRD--------ELLAQLHAAP 268
Tcfkh_tcas      ----HKSPSHSVDNSNSSEKKSSIQHGDDAHKTHHLDKNPEN--------TLGTMLSIHP 295
At.fkh_atep     ----QKCQKSPGDQSVKSEPEMNSSPKMDPKSSPMKVPEMEQPCLPPVNTSLPSTTDAYQ 270
fkh_pvul        ----HDDDPSCGMSNGQNSADSTPTSTGDGTPLSAPNTPPHP---------VEQSQMTQP 304
```

```
FoxA4a_xlae      PTSEQAGTASQLMYPLG-----------------------------------------LS 303
FoxA4b_xlae      PTSEQAGTASQLMYPLG-----------------------------------------LS 303
FoxA4_amex       ASDQRHSPGQQLFHNLG-----------------------------------------PA 320
FoxA3_olat       SSSHLHSPPVSLPPS--------------SSSLSLSGTSSSNPLLHPQSLASSSHLLPSP 364
UN_51_tnig       SSSHLHSTPVSMTASSVSSALA-------PSSLSLSASSSSNPLLHSQSLVGSPHLLPSA 373
FoxA3_drer       APNLLHSSPVPIPSS------------------VSASMPPSSSHLHSQGMGNSPHLLG-S 351
Foxa3_mmus       PSSTPYFSGLELPGELK------------------------------------------- 296
FoxA3_rnor       PSSAPYFTGLELPGELK------------------------------------------- 297
FOXA3_hsap       ASSTPYFTGLELPGELK------------------------------------------- 293
FoxA2_clal       LSPEHAAASPVSQGQHL-----------------------------------MSQHHSVL 326
HNF3b_omos       LSPEHTAASPVTQGQHL-----------------------------------MSQHHSVL 324
UN_45_tnig       LSPEHASASPVSQGQHL-----------------------------------MPPHHSVL 309
FoxA2_olat       LSPEHTAPSPVSQGQHL-----------------------------------MSQHHSVL 326
FoxA2_drer       LSPDHAAS-PTSQAQHL-----------------------------------LAQHHSVL 324
FoxA2_xtro       LSPDHAAS-PASQAQHL-----------------------------------LSQHHSVL 331
Foxa2_mmus       ASALSPPEPAPSPGQQQ----------------------------QAAAHLLGPPHHPGL 359
FoxA2_rnor       ASALSPPEPAPSPGQQQ----------------------------QAAAHLLGPPHHPGL 358
FOXA2_hsap       AAALSPPEPAPSPGQQQ----------------------------QAAAHLLGPPHHPGL 358
FoxA2_ggal       AGPSPGEPSAASP-----------------------------------AHHLLAPPHAGL 336
Foxa1_mmus       KSPASSSAPPISSGPGAL-----------------------------ASVPPSHPAHGLA 376
FoxA1_rnor       KSPASSSAPPISSGPGGW-----------------------------ICTPLSP--TWLA 374
FOXA1_hsap       KTPASSTAPPISSGPGAL-----------------------------ASVPASHPAHGLA 380
FoxA1b_xlae      AGPSPLSSHQNHS-------------------------------------------THSL 335
FoxA1_xtro       AGPSPLSSHQNHS-------------------------------------------THSL 336
UN_3_tnig        QLLSSLSLAP-----------------------------------------------HPM 286
AmHNF31_bflo     QQPQVQTTQPQDMQQHAQQHQGLP---------------------------ARPIPQQSS 318
HNF3_bflo        QPKPLAPTAVPVQSQHPQEQLGYP---------------------------TRPIPQTSP 346
SGF1_bmor        ELCLPEHTPLALEHYA-------------------------------------------- 284
Tcfkh_tcas       SKLDVEQMNLLHSNDLNMHQQHH-----QQNMSHEELSAMVNRCHPLSLSSDHQAMLHHN 350
At.fkh_atep      QMYQIQSFNANSNATNSSRNRLD-----CYKQEMLYSSHRYPESCSVSDPGMDHNAYQDI 325
fkh_pvul         KTEQPTHQNHSQAHVQ------------QQDMSHLTHSSQNQMGNCGTELTSMRQLHHDM 352

FoxA4a_xlae      NDGYLGLVGEDVHLKHDPFSGRHPFSITQLMSSEQDQ----------------TYANKME 347
FoxA4b_xlae      NDGYLGLVGEDVHLKHDPFSGRHPFSITQLMSSEQDQ----------------TYPNKME 347
FoxA4_amex       NDDFLSHLGAEAHVKPETLSLCHPFSITNLMSSEQQY-------------------HKMD 361
FoxA3_olat       MQQHMDLQSD-LKSLDPHYNFNHPFSITNLMSNEQKM-------------------DLKS 404
UN_51_tnig       MQHHMDLQSDPLKSLDPHYNFNHPFSITNLMSNEQKM-------------------DLKS 414
FoxA3_drer       PMHHLDLQNDPLKSMDPHFNFNHPFSITNLMSNEQKM-------------------DLKS 392
Foxa3_mmus       ---------------LDAPYNFNHPFSINNLMS--------------------------- 314
FoxA3_rnor       ---------------LDAPYNFNHPFSINNLMS--------------------------- 315
FOXA3_hsap       ---------------LDAPYNFNHPFSINNLMS--------------------------- 311
FoxA2_clal       AHEAH-------LKPEHHYSFNHPFSINNLMSSEQ-------------------QHHKMD 360
HNF3b_omos       AHEAH-------LKPEHHYSFNHPFSINNLMSSEQ-------------------QHHKMD 358
UN_45_tnig       AHEAH-------LKPEHHYSFNHPFSINNLMSSEQQ------------------HHHKMD 344
FoxA2_olat       AHEAH-------LKPEHHYSFNHPFSINNLMSSEQ-------------------QHHKMD 360
FoxA2_drer       AHEGH-------LKPEHHYSFNHPFSINNLMSSEQ-------------------QHHKMD 358
FoxA2_xtro       SHEAQSH-----LKPEHHYSFNHPFSINNLMSSEQQHH--------HHHHHNHHHHHKMD 378
Foxa2_mmus       PPEAH-------LKPEHHYAFNHPFSINNLMSSEQQ----------HHHSHHHHQPHKMD 402
FoxA2_rnor       PPEAH-------LKPEHHYAFNHPFSINNLMSSEQQ----------HHHSHHHHQPHKMD 401
FOXA2_hsap       PPEAH-------LKPEHHYAFNHPFSINNLMSSEQQ----------HHHSHHHHQPHKMD 401
FoxA2_ggal       PHDAH-------LKPEHHYAFNHPFSINNLMSSSEQQHH-------HHPHHHHHPSHKMD 382
Foxa1_mmus       PHESQLH-----LKGDPHYSFNHPFSINNLMSSSEQQ-------------------HKLD 412
FoxA1_rnor       PHESQLH-----LKGAPHYSFNHPFSINNLMSSSEQQ-------------------HKLD 410
FOXA1_hsap       PHESQLH-----LKGDPHYSFNHPFSINNLMSSSEQQ-------------------HKLD 416
FoxA1b_xlae      AHETHIH-----LKGDPHYSFNHPFSINNLMSSSEQQ-------------------HKLD 371
FoxA1_xtro       AHETHIH-----LKGDPHYSFNHPFSINNLMSSSEQQ-------------------HKLD 372
UN_3_tnig        AHESQLH-----LKGDPHYSFNHPFSINNLMSS-TEQ-------------------HKLD 321
AmHNF31_bflo     LPMSMGGYFSPEHLRAAHG-FTHPFSISNLMSQEHKP----------------------D 355
HNF3_bflo        MSAAMSMYSTDHIKTSVHPSFHHPFSINSIISQDHKLT---------------------E 385
SGF1_bmor        ----QLKQEP-----SGYAPAQHPFSITRLLPGADTKAD--------------------- 314
Tcfkh_tcas       PMSHHLKQEP-----SGFTSSNHPFSINRLLPTAESKAD--------------------- 384
At.fkh_atep      MKDLFFLQQPNYKLEPGFNATSHPFSINNIISNPDPKMD--------------------- 364
fkh_pvul         SMNHGLNLAPG--QLNHPHSFNHPFSITNLMS--ENKMD--------------------- 387
```

```
FoxA4a_xlae     MCPTTDHLVHYSNYS----SDYHNMASKNGLDMQTSSS-TDNGYYANMYSRPILSSL---- 399
FoxA4b_xlae     MCPTTDHLVHYSNYS----SDYHNLVSKNGLDMQTSSSSTDNGYYANMYSRPILSSL---- 400
FoxA4_amex      LRQAHEQMMHYSGYSSASPVDFHPMSGKPGLDMPSISS-DPGPYYTSMYSRPLLSSL---- 417
FoxA3_olat      YQDQVMAYNSYTAGSPVG--------AKQIYDSPGPAAMDSGAYYQTLYSRSVLNAS---- 453
UN_51_tnig      YQDQVMAYNSYAGSSPVA--------AKPIYDSSGPATMDSGTYYQTLYSRSVLNAS---- 463
FoxA3_drer      YQDQVMAYNSYATSSPVA--------AKQIYDNAGPSAIDSGAYYQTLYSRSVLNAS---- 441
Foxa3_mmus      ----------EQTSTPSK--------LDVGFGGYGAESGEPGVYYQSLYSRSLLNAS---- 353
FoxA3_rnor      ----------EQTSTPSK--------LDVGFGGYGAESGEPGVYYQSLYSRSLLNAS---- 354
FOXA3_hsap      ----------EQTPAPPK--------LDVGFGGYGAEGGEPGVYYQGLYSRSLLNAS---- 350
FoxA2_clal      LRTYEQVMHYSG-YGSPVTGALSMGSMTGKAGLDSSSI-PDTTYYQGVYSRPIMNSS---- 415
HNF3b_omos      LKTYEQVMHYSG-YGSPMAGPLSMGSMAGKAGLDSSSI-PDTTYYQGVYSRPIMNSS---- 413
UN_45_tnig      LKTYEQVMHYSG-YGSPMAGALSMGSMAGKHGLDSASI-PDTAYYQGVYSRPIMNSS---- 399
FoxA2_olat      LKTYEQVMHYSG-YGSPMTGALSMGSMAGKAGLDSASI-PDTSYYQGVYSRPIMNSS---- 415
FoxA2_drer      LKTYEQVMHYG--YGSPMAGTLSMGSMASKAGLDS----PDTSYYQGVYSRPILNSS---- 409
FoxA2_xtro      LKAYEQVMHYSG-YGSPMTGSLAMSTVTNKSGLESSPISSDTSYYQGVYSRPIMNSS---- 434
Foxa2_mmus      LKAYEQVMHYPGGYGSPMPGSLAMGPVTNKAGLDASPLAADTSYYQGVYSRPIMNSS---- 459
FoxA2_rnor      LKTYEQVMHYPGGYGSPMPGSLAMGPVTNKAGLDASPLAADTSYYQGVYSRPIMNSS---- 458
FOXA2_hsap      LKAYEQVMHYPG-YGSPMPGSLAMGPVTNKTGLDASPLAADTSYYQGVYSRPIMNSS---- 457
FoxA2_ggal      LKAYEQVMHYSG-YASPVPASLAMGPVTNKNPLESSPLAGETSYYQGVYSRPIMNSS---- 438
Foxa1_mmus      FKAYEQALQYSP-YGATLPASLPLGSASVATRSPIEPSALEPAYYQGVYSRPVLNTS---- 468
FoxA1_rnor      FKAYEQALQYSP-YGATLPASLPLGGASVATRSPIEPSALEPAYYQGVYSRPVLNTS---- 466
FOXA1_hsap      FKAYEQALQYSP-YGSTLPASLPLGSASVTTRSPIEPSALEPAYYQGVYSRPVLNTS---- 472
FoxA1b_xlae     FKAYEQALQQYS-SYGGGLQGMPLGSPSMTGRGTIEPSALEPTYYQGVYSRPVLNTS---- 427
FoxA1_xtro      FKAYEQALQQYS-SYSGGLPGMPLGSPSMAGRGSIEPSALEPTYYQGVYSRPVLNTS---- 428
UN_3_tnig       LKAYEALQYSSY-STG-----------GPSGLGGRSMESLEATYYQGVYPRPLLNTS---- 366
AmHNF31_bflo    LKEYAAMGYSGYNSMS-------PTGVPKTTMSMDS---MGTDYYQGYVPQHSQPSSL--- 403
HNF3_bflo       LKGYDPMQYSGYASMYNT----NPSVVPKQEMETPASTEPATGYFSGYVPQYSTTASTLQS 442
SGF1_bmor       LKMYDVNYGYGH--------------SPADNYYQSPLYHHHHAHAQPPL------------ 349
Tcfkh_tcas      IKMYADMHQYGYNTLSPLPSSVHSHSTIGNDYYNSPLYHTSAGTTSL-------------- 431
At.fkh_atep     SKFFEMTVPHYSNYSSGMP-------TSDNMAYYSPSFYSVPQPVTSDV------------ 406
fkh_pvul        FKMYEAISGYGAYTQMSPMS-MPKEASPPMNAQDGSYYKTYAPHSTASL------------ 435
```

B. Alignment of the FoxD cluster of sequences.

Conserved and functionally important regions noted in the literature are highlighted. Green: forkhead domain (NCBI Protein database, see Additional file 1 for accession numbers) Red Box: EH1 motif [4, 5, 6] Positively selected sites identified by branch-site analysis are indicated by purple boxes.

```
FoxD5_drer              ----------MTLSQDYEQVQRTPISPEDDEIDIVGGDHSDS------------------ 32
FoxD5_omos              ----------MTLSSEFEASQHAGLPIQEDAIDIVGEDVHYRNE---------------- 34
xfd12_xlae              ------MSFSQESGAHHHPQDYAGLSDEEDEIDILGEDDPCSLKSHFYLQPTHS------ 48
xfd12prime_xlae         ------MSFSQESGTHHNSLDYAGVSDEEDEIDILGEDDPCSLKSHFYLQPTHS------ 48
xfd12dblprime_xlae      ------MNLSQDSSAHHQSQDYAGVSDDEDEIDILGEDDPCSPRSHIYQQPTDS------ 48
FoxD3_drer              -------MTLSGGTSASNMSGQTVLTADDVDIDVVGEGDEGMEQDSDCESQCMQ------ 47
UN_48_tnig              ------MTLSGGSERASDMSGQTVLTAEDVDIDVVEG----------------------- 31
FoxD3b_xlae             -------MTLSSSGSASDMSGQTVLSADDADIDVVGEGDEALDKDSECESPVG------- 46
xfd6_xlae               -------MTLSGSGSASDMSGQTVLSADDADIDVVGEGDEALDKDSECESTAG------- 46
FOXD3_hsap              -------MTLSGGGSASDMSGQTVLTAEDVDIDVVGEGDDGLEEKDSDAGCDSP------ 47
Foxd3_mmus              -------MTLSGSGSASDMSGQTVLTAEDVDIDVVGEGDDGLEEKDSDAGCDSP------ 47
FoxD3_ggal              ---------MTLSGGGSDMSGQTALAAEDVDIDVVGEGDDAPGKDGDGEARSPA------ 45
FoxD2_ggal              MTLGSSGGGGCGIMSERSPEEEPLSEVEDADIDVVGPPQDGAKYSE-------------- 46
FoxD2_xlae              -------------MTLGTEMSDNSLLSEDTDIDVVGDMGAKDGKYS-------------- 33
FOXD2_hsap              -----MTLGSCCCEIMSSESSPAALSEADADIDVVGGGSGGGELPA-------------- 41
Foxd2_mmus              -----MTLGSCCCEIMSSESSPAALSEPDADIDVVGGGSGGGELTA-------------- 41
FOXD1_hsap              -------------MTLSTEMSDASGLAEETDIDVVGEGEDEEDEEE-------------E 34
Foxd1_mmus              -------------MTLSTEMSDASGLAEETDIDVVGEGEDDEEEED-------------D 34
FoxD1_ggal              -------------MTLSSEMSEASALAEETDIDVVGEEDDEEDEEEPQPRHRRRRRSYAE 47
FoxD4_ggor              -----MNLPRAERLRSTPQRSLRDSDGEDDKIDVLGEEEDEDEVEDEEE----------- 44
FoxD4_ptro              -----MNLPRAERLRSTPQRSLRDSDGEDGKIDVLGEEED----EDEEE----------- 40
FOXD4L2_hsap            -----MNLPRAERLRSTPQRSLRDSDGEDGKIDVLGEEEDEDEVEDEEE----------- 44
FOXD4L3_hsap            -----MNLPRAERPRSTPQRSLRDSDGEDGKIDVLGEEEDEDEVEDEEE----------- 44
FOXD4b_hsap             -----MNLPRAERPRSTPQRSLRDSDGEDGKIDVLGEEEDEDEVEDEEE----------- 44


FoxD5_drer              ---------EREYFMRDPTEVDHSGSES-------------------------------- 51
FoxD5_omos              --------CSTGSSAESGAEFDSSEPES-------------------------------- 54
xfd12_xlae              ------VMGDSEMLSPSKLSCTESESDS-------------------------------- 70
xfd12prime_xlae         ------DMGDSGMLSPSKLSCTESESDS-------------------------------- 70
xfd12dblprime_xlae      ------DMGDRGVLSPSKLSCNESASHS-------------------------------- 70
FoxD3_drer              --DRGDEVEEIEVKERSTSPCESN------------------------------------ 69
UN_48_tnig              -------------APCCESSGEG------------------------------------- 41
FoxD3b_xlae             ---HHDEVDALGGKEIPRSPSGSS------------------------------------ 67
xfd6_xlae               ---HTDEVGELGGKEIPRSPSGSG------------------------------------ 67
FOXD3_hsap              --AGPPELRLDEADEVPPAAPHHGQPQPPHQQPLTLPKEAAGAGAGPGGDVGAPEADGCK 105
Foxd3_mmus              --AGPPDLRLDEADEGPPVSAHHGQSQP-------QALALPTEATGPGNDTGAPEADGCK 98
FoxD3_ggal              --ALPLPLDEAAEPGEPERAARRAAAAR----------------------QPGPGRPEGG 81
FoxD2_ggal              --------DEEE-----------DDDDEEDDEEEGGGPWGSPAADGGPPSAHGGVPERLS 87
FoxD2_xlae              --------DYHS-----------DNDSDDN------------------------------ 44
FOXD2_hsap              --------RSGPRAPRDVLPHGHEPPAEEAEADLAEDEEESGGCSDGEPRALASRGAAAA 93
Foxd2_mmus              --------RSGPRAPRDVLPHGHEPPPEEAEADVAEDEEESGGCSDCEPRALAPRGAAAA 93
FOXD1_hsap              DDDEGGGGGPRLAVPAQRRRRRRSYAGEDELEDLEEEEDDDDILLAPPAGGSPAPPGPAP 94
Foxd1_mmus              DDEGGGGRGGGGSRLPSSAQRRRRSYAGEDDLEDLEEEDDDDLLLASRPAASPAPPGPAP 94
FoxD1_ggal              DEEEEEEEEEEDAGDLHDDALLPRSPVRAGGGGGGGGGGGAGGGDGPGGSRPPSRGGPQ 107
FoxD4_ggor              --------EASQQFLEQSLQQGLQVARWSG------------------------VALPRE 72
FoxD4_ptro              --------EASQQFLEQSLQPGLQVARWGG------------------------VALPRE 68
FOXD4L2_hsap            --------EASQQFLEQSLQPGLQVARWGG------------------------VALPRE 72
FOXD4L3_hsap            --------AARQQFLEQSLQPGLQVARWGG------------------------VALPRE 72
FOXD4b_hsap             --------EARQQFLEQSLQPGLQVARWGG------------------------VALPRE 72
```

```
FoxD5_drer              ---------SGESE---SDFASSTVAPK--QSSSVKPPYSYIALITMAILQSPMKKLTLS 97
FoxD5_omos              ---------SGESE---NSFCADAPPSRKAQSSSVKPPYSYIALITMAILQSPLKKLTLS 102
xfd12_xlae              ---------SGESEGGTSKDSSTTPTGSKAKRTLVKPPYSYIALITMAILQSPHKKLTLS 121
xfd12prime_xlae         ---------SGESEGGTSKDSPATSPGGKAKRALVKPPYSYIALITMAILQSPHKKLTLS 121
xfd12dblprime_xlae      ---------SGERERGTSKHSLDTTTNGKVKRALVKPPYSYIALITIAIMQSPHKKLTLS 121
FoxD3_drer              ---------ADGETKGDAQESSTGPMQNKPKSSLVKPPYSYIALITMAILQSPQKKLTLS 120
UN_48_tnig              ---------DTGKGEGQEQGARRVGGIQKPKNSLVKPPYSYIALITMAILQSPQKKLTLS 92
FoxD3b_xlae             ---------TDAEGKGESQQQQQEGIQNKPKNSVVKPPYSYIALITMSILQSPQKKLTLS 118
xfd6_xlae               ---------TEAEGKGESQQQQQEGIQNKPKNSLVKPPYSYIALITMSILQSPQKKLTLS 118
FOXD3_hsap              GGVGGEEGGASGGGPGAGSGSAGGLAPSKPKNSLVKPPYSYIALITMAILQSPQKKLTLS 165
Foxd3_mmus              G---GEDAVTGGGGPGAGSGATGGLTPNKPKNSLVKPPYSYIALITMAILQSPQKKLTLS 155
FoxD3_ggal              RGGGGGGGGGEEGASGGGAAAAAAAGQSKPKSSLVKPPYSYIALITMAILQSPQKKLTLS 141
FoxD2_ggal              PAGARSPRAPGPRPGKRAAGGGGGGGGGGGKNPLVKPPYSYIALITMAILQSPKKRLTLS 147
FoxD2_xlae              --VARTPRGDPASPDLSSGSESNQRAEKSPKNALVKPPYSYIALITMSILQSPKKRLTLS 102
FOXD2_hsap              AGSPGPGAAAARGAAGPGPGP--PSGGAATRSPLVKPPYSYIALITMAILQSPKKRLTLS 151
Foxd2_mmus              AGSPGPGVQAARGATGPGPGPGPPSGGAATRSPLVKPPYSYIALITMAILQSPKKRLTLS 153
FOXD1_hsap              AAGAGAG-----GGGGGGGAGGGGSAGSGAKNPLVKPPYSYIALITMAILQSPKKRLTLS 149
Foxd1_mmus              APGTGSGGCSGAGAGGGAGGGTGAGTGGGAKNPLVKPPYSYIALITMAILQSPKKRLTLS 154
FoxD1_ggal              KAAAAGGGGAGGGGGGGGGAGGGGGGGGGGKNSLVKPPYSYIALITMAILQSPKKRLTLS 167
FoxD4_ggor              HIEGGGGPSDPSEFGTKFRAPPRSAAASEDARQPAKPPYSYIALITMAILQNPHKRLTLS 132
FoxD4_ptro              HIEGGGGPSDPSEFSTKFRAPPRSAAASEDARQPAKPPYSYIALITMAILQNPHKRLTLS 128
FOXD4L2_hsap            HIEGGGGPSDPSEFGTKFRAPPRSAAASEDARQPAKPPYSYIALITMAILQNPHKRLTLS 132
FOXD4L3_hsap            HIEGGGGPSDPSEFGTKFRAPPRSAAASEDARQPAKPPYSYIALITMAILQNPHKRLTLS 132
FOXD4b_hsap             HIEGGGGPSDPSEFGTKFRAPPRSAAASEDARQPAKPPYSYIALITMAILQNPHKRLTLS 132

FoxD5_drer              GICDFISNKFPYYKEKFPAWQNSIRHNLSLNDCFIKIPREPGNPGKGNYWSLDPASEDMF 157
FoxD5_omos              GICDFISNKFPYYRDKFPAWQNSIRHNLSLNDCFIKIPREPGNPGKGNYWSLDPASEDMF 162
xfd12_xlae              GICDFISSKFPYYKDKFPAWQNSIRHNLSLNDCFIKIPREPGNPGKGNYWTLDPASEDMF 181
xfd12prime_xlae         GICDFISSKFPYYKDKFPAWQNSIRHNLSLNDCFIKIPREPGNPGKGNYWTLDPASEDMF 181
xfd12dblprime_xlae      GICDFISSKFPYYKDKFPAWQNSIRHNLSLNDCFIKIPREPGNPGKGNYWTLDPASKDMF 181
FoxD3_drer              GICEFISNRFPYYREKFPAWQNSIRHNLSLNDCFVKIPREPGNPGKGNYWTLDPQSEDMF 180
UN_48_tnig              GICEFISSRFPYYREKFPAWQNSIRHNLSLNDCFVKIPREPGNPGKGNYWTMDPASEDMF 152
FoxD3b_xlae             GICEFISSRFPYYREKFPAWQNSIRHNLSLNDCFIKIPREPGNPGKGNYWTLDPQSEDMF 178
xfd6_xlae               GICEFISNRFPYYREKFPAWQNSIRHNLSLNDCFVKIPREPGNPGKGNYWTLDPQSEDMF 178
FOXD3_hsap              GICEFISNRFPYYREKFPAWQNSIRHNLSLNDCFVKIPREPGNPGKGNYWTLDPQSEDMF 225
Foxd3_mmus              GICEFISNRFPYYREKFPAWQNSIRHNLSLNDCFVKIPREPGNPGKGNYWTLDPQSEDMF 215
FoxD3_ggal              GICEFISNRFPYYREKFPAWQNSIRHNLSLNDCFVKIPREPGNPGKGNYWTLDPQSEDMF 201
FoxD2_ggal              EICEFISGRFPYYREKFPAWQNSIRHNLSLNDCFVKIPREPGNPGKGNYWTLDPESADMF 207
FoxD2_xlae              EICEFISNRFPYYREKFPAWQNSIRHNLSLNDCFVKIPREPGNPGKGNYWTLDPESADMF 162
FOXD2_hsap              EICEFISGRFPYYREKFPAWQNSIRHNLSLNDCFVKIPREPGNPGKGNYWTLDPESADMF 211
Foxd2_mmus              EICEFISGRFPYYREKFPAWQNSIRHNLSLNDCFVKIPREPGNPGKGNYWTLDPESADMF 213
FOXD1_hsap              EICEFISGRFPYYREKFPAWQNSIRHNLSLNDCFVKIPREPGNPGKGNYWTLDPESADMF 209
Foxd1_mmus              EICEFISSRFPYYREKFPAWQNSIRHNLSLNDCFVKIPREPGNPGKGNYWTLDPESADMF 214
FoxD1_ggal              EICEFISGRFPYYREKFPAWQNSIRHNLSLNDCFVKIPREPGNPGKGNYWTLDPESADMF 227
FoxD4_ggor              GICAFISGRFPYYRRKFPAWQNSIHHNLSLNDCFFKIPREPGHPGRGNYWSLDPASQDMF 192
FoxD4_ptro              GICAFISGRFPYYRRKFPAWQNSIHHNLSLNDCFVKIPREPGHPGKGNYWSLDPASQDMF 188
FOXD4L2_hsap            GICAFISGRFPYYRRKFPAWQNSIRHNLSLNDCFVKIPREPGHPGKGNYWSLDPASQDMF 192
FOXD4L3_hsap            GICAFISGRFPYYRRKFPAWQNSIRHNLSLNDCFVKIPREPGHPGKGNYWSLDPASQDMF 192
FOXD4b_hsap             GICAFISGRFPYYRRKFPAWQNSIRHNLSLNDCFVKIPREPGHPGKGNYWSLDPASQDMF 192
```

```
FoxD5_drer              DNGSFLRRRKRFKRNQP-------EFTKDSLVLYHPTLSYRAYGR--------------- 195
FoxD5_omos              DNGSFLRRRKRFKRNQP-------EFGKDGLMFYSSLNCYRPYGH--------------- 200
xfd12_xlae              DNGSFLRRRKRFKRHQQ-------EFFKDGLMMYNSLPYYRPY----------------- 217
xfd12prime_xlae         DNGSFLRRRKRFKRHQQ-------EFFKDGLMMYNSLPYYRPY----------------- 217
xfd12dblprime_xlae      DNGSFLRRRKRFKRQHQ-------ELFKDGLVMYNPLHYCTPN----------------- 217
FoxD3_drer              DNGSFLRRRKRFKRHQPDILRDQTALMMQSFGAYGIGNPYGRHYG--------------- 225
UN_48_tnig              DNGSFLRRRKRFKRVQPDMLRDQTALMMQSFGAYSLGGPYGRHYG--------------- 197
FoxD3b_xlae             DNGSFLRRRKRFKRQQQDTLREQTALMMQSFGAYSLASPYGRHYG--------------- 223
xfd6_xlae               DNGSFLRRRKRFKRQQQDSLREQTALMMQSFGAYSLASPYGRHYG--------------- 223
FOXD3_hsap              DNGSFLRRRKRFKRHQQEHLREQTALMMQSFGAYSLAAAAGAAGPYGRPYGLHP------ 279
Foxd3_mmus              DNGSFLRRRKRFKRHQQEHLREQTALMMQSFGAYSLAAAAGAG-----PYGLHP------ 264
FoxD3_ggal              DNGSFLRRRKRFKRHQQEHLRDQTALMMQGFGAYGLAGPYGRPYG--------------- 246
FoxD2_ggal              DNGSFLRRRKRFKR----------HEQPDPPHPELLLRAGARSRRLPARLR--------- 248
FoxD2_xlae              DNGSFLRRRKRFKR----------QQSNEILR--------DPSSFMPAAFG--------- 195
FOXD2_hsap              DNGSFLRRRKRFKRQPLPPPHPHPHPHPELLLRGGAAAAGDPGAFLPGFAA--------- 262
Foxd2_mmus              DNGSFLRRRKRFKRQPLPPPHPHPHPHPELLLRGGAAAAGDPGAFLSSFAA--------- 264
FOXD1_hsap              DNGSFLRRRKRFKRQPLLPPNAAAAESLLLRGAGAAGGAGDPAAAAALFPPAPPPPPHAY 269
Foxd1_mmus              DNGSFLRRRKRFKRQPLLAP-HAAAEALLLRGAGPAAGAGDPGAALFPPPPPPP----AC 269
FoxD1_ggal              DNGSFLRRRKRFKRQQLPAP------ELLLRAVDPAAFLPQPPPQPPQQP--------PC 273
FoxD4_ggor              DNGSFLRRRKRFKRHQLTPG----AHLPHPFPLPAAHAALHNPRP--------------- 233
FoxD4_ptro              DNGSFLRRRKRFKRHQLTPG----AHLPHPFPLSAAHAALHNPRP--------------- 229
FOXD4L2_hsap            DNGSFLRRRKRFKRHQLTPG----AHLPHPFPLPAAHAALHNPRP--------------- 233
FOXD4L3_hsap            DNGSFLRRRKRFKRHQLTPG----AHLPHPFPLPAAHAALHNPRP--------------- 233
FOXD4b_hsap             DNGSFLRRRKRFKRHQLTPG----AHLPHPFPLPAAHAALHNPHP--------------- 233

FoxD5_drer              -----------PYCVSGAVP-AQTNPVGYLPVPDGIMVPPP------------------- 224
FoxD5_omos              -----------PYSITGQVSPTAASSVRYMPLQESIVMPSS------------------- 230
xfd12_xlae              -------------SAIQPQPVLQQTSLTCMAIPETLPMSTH------------------- 245
xfd12prime_xlae         -------------SALQPQPMLQQTPLACMAIPETLSMPTN------------------- 245
xfd12dblprime_xlae      -------------SALQAQ----QIPMTCLAIPENFAMPNH------------------- 241
FoxD3_drer              ----------IHPAAYTHPAALQYPYIPP-VGPMLPPAVPLLP----------------- 257
UN_48_tnig              ----------IHPAAYSHPAALQYPYIPP-VGHMLPPGVPLLP----------------- 229
FoxD3b_xlae             ----------LHPAAYTHPAALQYPYIPP-VGPMLPPAVPLLP----------------- 255
xfd6_xlae               ----------LHPAAYTHPAALQYPYIPP-VGHMLPPAVPLLP----------------- 255
FOXD3_hsap              AAAAGAYSHPAAAAAAAAAAALQYPYALPPVAPVLPPAVPLLP----------------- 322
Foxd3_mmus              AAAAGAYSHPAAAAAAAAAAALQYPYALPPVAPVLPPAVPLLP----------------- 307
FoxD3_ggal              ----------LPPGAYPHPAALQYPYIPP-VGPMLPPACPLLP----------------- 278
FoxD2_ggal              -YGPYGYNYGLQLQGLPPGPPAPPPPPRRRLRGAFPFSAP-------------------- 288
FoxD2_xlae              -YGPYGYNYGLQLHNYQQHP------------GATFSFQPS------------------- 223
FOXD2_hsap              -YGAYGYGYGLALPAYGAPPPGPAPHPHPHPHAFAFAAAAAAAPCQLSVPPGRAAAPPPG 321
Foxd2_mmus              -YGAYGYGYGLALPAYGAPPPGPAPHPHPHPHAFAFAATAP---CQLSVPPGRAAAPPPG 320
FOXD1_hsap              GYGPYGCGYGLQLPPYAPPSALFAAAAAAAAAAAFHPHSP-------------------- 309
Foxd1_mmus              GYGAYGCAYGLQLPPCAPPSALFAAAAAAAAAAAFHPHSPP------------------- 309
FoxD1_ggal              AYGPYGCGYGLQLQLPQYHPHSALFAFHHPSPPPRQPPAAPAG----------------- 314
FoxD4_ggor              ---------GPLLGAPAPPQPVPGAYPNTAPGRRPYALLHPHP----------------- 267
FoxD4_ptro              ---------GPLLGAPAPPQPVPGAYPNTAPGRRPYALLHPHP----------------- 263
FOXD4L2_hsap            ---------GPLLGAPAPPQPVPGAYPNTAPGRRPYALLHPHP----------------- 267
FOXD4L3_hsap            ---------GPLLGAPAPPQPVPGAYPNTAPGRRPYALLHPHP----------------- 267
FOXD4b_hsap             ---------GPLLGAPAPPQPVPGAYPNTAPGRRPYALLHPHP----------------- 267
```

```
FoxD5_drer             ----------FFQYQTMNIKIH--------------------------DAPEIQQRPEHK 248
FoxD5_omos             ----------SYHLLPQPLNSHGKCVGPKDFRAQ-------------LCAAEPAEPKSGP 267
xfd12_xlae             ----------LAPYPDIKRKVSYP---AQGVHRG-------------FKAQDADNHPNNS 279
xfd12prime_xlae        ----------LTPYPDIKRKAHYP---DQGAHRG-------------FEGQDANNHPNKS 279
xfd12dblprime_xlae     ----------LVPYPDINITVPCP---DQGVHRV-------------LTAQDVDNHPSNS 275
FoxD3_drer             ----------SAELNRKAFSS--QLSPSLQLQLN------------SLS--TASIIKSEP 291
UN_48_tnig             ----------SAELNRKAFNS--QLSPSLQLQLN------------SLS--TASMIKSEP 263
FoxD3b_xlae            ----------SSELTRKAFSS--QLSPSLQLQLS------------SLSSTAASIIKSEP 291
xfd6_xlae              ----------SSELTRKAFSS--QLSPSLQLQLS------------SLSSTAASIIKSEP 291
FOXD3_hsap             ----------SGELGRKAAAFGSQLGPGLQLQLNSLGAAAAAAGTAGAAGTTASLIKSEP 372
Foxd3_mmus             ----------SGELGRKAAAFGSQLGPSLQLQLNTLGAAAAAAGTAGAAG-TTSLIKSEP 356
FoxD3_ggal             ----------SGELSRKAFNA--QLGPSLQLQLS-------------SLGAAGSIVKSEP 313
FoxD2_ggal             ----------HCPLPVGPPSAASVFSAASGLPSFLGGELNCRKSFYHPQLSPTAL----- 333
FoxD2_xlae             ----------HCPLPP-----PASVFSSPTLSPFLGNELSRKS-----LYSQLSP----- 258
FOXD2_hsap             PPTASVFAGAGSAPAPAPASGSGPGPGPAGLPAFLGAELGCAKAFYPASLSPPAAGTAAG 381
Foxd2_mmus             PPTASVFASAASAPAPAPAPGSGPSP--AGLPAFLGAELGCAKAFYPASLSPPAAGTAAS 378
FOXD1_hsap             ----------PPPPPP---HGAAAELARTAFGYRPHPLGAALPGPLPASAAKAGGPGASA 356
Foxd1_mmus             ----------PPPPPPPPPPGAAAELARTAFGYRSHALAAALPGPLQAAAVKAGGRGAAA 359
FoxD1_ggal             ---APAAALPPPPPPPPPPRRRAPLLPAAELARTPFGYPHPLGPALAASLHAAKPGSGAA 371
FoxD4_ggor             ---------LRYLLLSAPAYAGAPKKAEGADLATPAPFPCCSPHLVLSLGRRARVWRRHR 318
FoxD4_ptro             ---------LRYLLLSAPAYAGAPKKAEGADLATPAPFPCCSPHLVLSLGRRARVWRRHR 314
FOXD4L2_hsap           ---------LRYLLLSAPVYAGAPKKAEGADLATPAPFPCCSPHLVLSLGRRARVWRRHR 318
FOXD4L3_hsap           ---------LRYLLLSAPVYAGAPKKAEGADLATPAPFPCCSPHLVLSLGRRARVWRRHR 318
FOXD4b_hsap            ---------LRYLLLSARVYAAAPKKAEGADLATPAPFPCCSPHLVLSLGRRARVWRRHR 318

FoxD5_drer             TQRCSFSIDSIMAKSTESS------------------------SKSSAHHLTPDYSFVFP 284
FoxD5_omos             QAKCSFSIDSIMSRPSSIS------------------------QHNSNPQLGPHGALGYG 303
xfd12_xlae             QSKCSFSIENIMRKPKEPE------------------------PNIQSFNSHWNYN-HVF 314
xfd12prime_xlae        QSKCSFSIENIMKKPKEPE------------------------PSFPSFNSHWNYNNHLL 315
xfd12dblprime_xlae     HSKCSFSIENIMGETKEPE------------------------KHLTSFNQNWNYN-HLL 310
FoxD3_drer             SSRPSFSIENIIGVSSSLR-----------------------AIQTFLRPPVTVQSALLS 328
UN_48_tnig             SNRPSFSIENIIGVSSASSSS-------------------PGAAQAFLRPPVTVQSALLS 304
FoxD3b_xlae            SSRPSFSIENIIGVSAASS----------------------VAPQTFLRPPVTVQSALMS 329
xfd6_xlae              SSRPSFSIENIIGVSAASS----------------------AAPHTFLRPPVTVQSALMS 329
FOXD3_hsap             SARPSFSIENIIGGGPAAPGGSAVGAG---VAGGTGGSGGGSTAQSFLRPPGTVQSAALM 429
Foxd3_mmus             SARPSFSIENIIGAGSAAPGGSAGGGGSGGGAGGGGGGSGGGGGAQSFLRPPGTVQSAALM 416
FoxD3_ggal             SSRPSFSIENIIGGPAASSAP---------------------SAQTFLRPPVTVQSGLVA 352
FoxD2_ggal             --PAALLQTLKPDPTPAGTGGTAAATTNPSRPSFSIDNIIGGAVPPPPSTNPSAAPAAPY 391
FoxD2_xlae             --TLPILHTLKPDAQ---------------SRPSFSIDNIIGGSGSTPSPTSPYTAQPGTH 302
FOXD2_hsap             LPTALLRQGLKTDAGGGAGGGGAGAGQRPSFSIDHIMGHGGGGAAPPGAGEGSPGPPFAA 441
Foxd2_mmus             LSTALLRQGLKTDAGGGAGGGGAGTGQRPSFSIDHIMGHGGGGAAPPGSGDGSPGSPFAA 438
FOXD1_hsap             LARSPFSIESIIGGSLGPAAAAAAAAQAAAAAQASPSPSPVAAPPAPGSSGGGCAAQAAV 416
Foxd1_mmus             LARSPFSIESLIGRTRGPAAAGAHVSSGAASGTAP------------GPGGGGCAVQAAA 407
FoxD1_ggal             VARSPFSIESIIGGGPGPGLGAGPAPGAGGSCASQ------------------------- 406
FoxD4_ggor             EADASLSSALRVLCKGSGERVQGLRRVCP-------------RPRGATATCSSDHQACCIP 365
FoxD4_ptro             EADASLSSALRVLCKGSGERVQGLRRVCP-------------RPRGATATCSSDHQACCIP 361
FOXD4L2_hsap           EADASLSSALRVLCKGSGERVQGLRRVCP-------------RPRGATATCSSDHQACCIP 365
FOXD4L3_hsap           EADASLSSALRVLCKGSGERVQGLRRVCP-------------RPRGATATCSSDHQACCIP 365
FOXD4b_hsap            EADASLSSALRVLCKGSGERVQGLRRVCP-------------RPRGATATCSSDHQACCIP 365
```

```
FoxD5_drer              RPTT---SCVAPSLVPVPTR----TPLLKTVPFS-ETLRMVYPHC--------- 321
FoxD5_omos              QLMPGPAACLVPTLLQPSRNQFCPPPILSTAPFINEHLRLSYPRC--------- 348
xfd12_xlae              QRPS--------SCLLPAVLNLSTGPLLANTQGARQYNLIQFPGCY-------- 352
xfd12prime_xlae         QRPS--------SCFLPAVLNLSTGPLLANVQGTRQYNLIKFPGSY-------- 353
xfd12dblprime_xlae      QSSR--------LCLLPSGS------HLANAHHSAQCNLIKFPGCY-------- 342
FoxD3_drer              --AQSLSLTRTSAAIAPILSVPSNIISGQFLPTASTAAVSKWPSQ--------- 371
UN_48_tnig              --AQSLSLTRTSAAIAPILSVPSSIISGHVLPAATAAAVSKWPSQ--------- 347
FoxD3b_xlae             --HQPLVLSRSTAAIGPILSVPTNLISGQFLPTAATAVAKWPAQ---------- 371
xfd6_xlae               --HQPLALSRSTAAIGPILSVPTNLISGQFLPTAAAAVAKWPAQ---------- 371
FOXD3_hsap              ATHQPLSLSRTTATIAPILSVPLSGQFLQPAASAAAAAAAAAQAKWPAQ----- 478
Foxd3_mmus              ATHQPLSLSRTTATIAPILSVPLSGQFLQPAASAAAAAAAAVQAKWPAQ----- 465
FoxD3_ggal              --HQPLALARTTAAIAPILSVPTNIIAGQFLQPPAAVQAKWPAQ---------- 394
FoxD2_ggal              PSGQAGPPAQLLAVLSPALAPSQPHGGLAHEP--LLQPAQNFSAKITNVGSCHF 443
FoxD2_xlae              PP--------VIAMLSPSLAPMHNHLNLAHEN--LLPPGQNFSSKITNLNSCHF 346
FOXD2_hsap              AAGPGGQAQVLAMLTAPALAPVAGHIRLSHPGDALLSSGSRFASKVAGLSGCHF 495
Foxd2_mmus              AAGPGGQAQVLAMLTAPALTPVAGHIRLSHPGDSLLSSGPSFASKVAGLSGCHF 492
FOXD1_hsap              GPAAALTRSLVAAAAAAASSVSSSAALGTLHQGTALSSVENFTARISNC----- 465
Foxd1_mmus              GPAVALTRSLVVAAAAAASSVSSSAALGTLHQGTALSSVENFTARISNC----- 456
FoxD1_ggal              ---------------SGAATGLSRSLGSGLAPAAALPAAPGLAARISNC----- 440
FoxD4_ggor              KPLPLCCKCPPPPLLGQFCSNSSSIRRRTAPTAALPPRARCWAGTCQPRRRC-- 417
FoxD4_ptro              KPLPLCCKCPPPPLLGQFCSNSSSIRRRTAPTAALPPRARCWAGTCRPRRRC-- 413
FOXD4L2_hsap            KPLPLCCKCPPPPLLGQFCSNSSSIRRRTAPTAALPPRARCWAGTCRPRRRC-- 417
FOXD4L3_hsap            KPLPLCCKCPPPPLLGQFCSNSSSIRRRTAPTAALPPRARCWAGTCRPRRRC-- 417
FOXD4b_hsap             KPLPLCCKCPPPLLLGQFCSNSSSIRR-TAPTAALPPRARCWAGTCRPRRRC-- 416
```

C. Alignment of the FoxI cluster of sequences.

Conserved and functionally important regions noted in the literature are highlighted. Green: forkhead domain (NCBI protein database, see Additional file 1 for accession numbers) Yellow: transactivation domain [7] Red Box: EH1 motif [5]

```
FOXI1_hsap      --------MSSFDLP----APSPPRCSPQFPSIGQEPPEMNLYYENF--FHPQGVPSPQR 46
Foxi1_mmus      --------MSSFDLP----APSPPRCSPQFPSIGQEPPEMNLYYENF--FHPQGMPSPQR 46
FoxI1_xlae      --------MSAFDPQ----AHSPPRCGPQFPSIGQEPPEMNIYCESF--LHPQTMPSPQR 46
FoxI3b_drer     --------MTSYESQ----GQSPTRCGPQFLSLGQEPPELSLYSDSY--YPPPSLPSPQR 46
UN_46_tnig      --------MSSFEAQ----GQSPPRCGPQFPSLGQEPPELSMYSDCY--YPPPSLPSPQR 46
FoxI3a_drer     --------MTSFVPQ----SLSP-----QFHSMGQESQEFSLYGDNF--YSAQHVPSPQQ 41
FoxI2_drer      --------MNTIDAQIHSNNNAAVNHLQQLPKSAHETSDMAVYCDNFSVYHQQSLPAAQR 52
FoxI1c_xlae     --------MNSIHLPSNQRTSASSLH-QHHPKGAQEASEMAVYCDNFSMYHQQNLHSSQR 51
FoxI1_drer      MFLEGERIMNAFGQQPSSQQTSPLQQQDILDMTVYCDSNFSMYQQNLHHHHHHHHHQRPP 60
Foxi2_mmus      --------M-SFSTEPP----APAQAGGELDMAGFCDS--------LGSCSVPHGLTRAI 39

FOXI1_hsap      --P-SFEGGGEYGATPNPYLWFNGPTMT-PPPYLP-----GPN-ASPFLPQAYGVQRP-L 95
Foxi1_mmus      --PTSFEGGGEYGTTPNPYLWFNGPAMT-PPPYLP-----GTN-ASPFLPQAYGMQRQ-L 96
FoxI1_xlae      --PSNFETG-DYSTTANPYLWLNGPSIT-PPPYLP-----GSN-SSHFMPQAYGMQRQ-L 95
FoxI3b_drer     TNPSSYELGDYAASSPNPYLWFNSPGMN-SAPYLG--GTPGPA-GPSFVPQHYGMQRPYL 102
UN_46_tnig      TTPTSYDLNDYATSSPNPYLWFNGSGIN-TSPYLATTGAPGNA-SSPFIPQHYGMQRSYL 104
FoxI3a_drer     TLPSAYDFGEYAGQTSNPYLWFNGPGLS-PAPCLT----TG--------PQHYGMAKQYV 88
FoxI2_drer      --PAGYGLGDYATP--NPYLWLNGPGVNSSSSYIH-----GNN-SPSFIPPAYGSQRQYL 102
FoxI1c_xlae     --APNYGIGDYAPPT-NPYLWLGGPGVSNSSSYLH-----GNN-PTSFMSPSYGSQRQFL 102
FoxI1_drer      AHPSSYGLGEYSSPSTNPYLWMNSPGIT-STPYLS-----SPN-GGSYIQSGFGSNQRQF 113
Foxi2_mmus      AHPPSYGRTDLSSGRR---LWVNSAALS-PAPYAT-----GPGPAPSYAAATLAVPG-SL 89

FOXI1_hsap      LPSVSGLGGSDLGWLPIPSQEELMKLVRPPYSYSALIAMAIHGAPDKRLTLSQIYQYVAD 155
Foxi1_mmus      LP-------SDLGWLPIPSQEELMKLVRPPYSYSALIAMAIHGAPDQRLTLSQIYQYVAD 149
FoxI1_xlae      LPNMHGLGSSELGWLPIPSQEELMKLVRPPYSYSALIAMAIHGAPDKRLTLSQIYQYVAD 155
FoxI3b_drer     GPGPPGGPGGELSWFSMPSQEDLMKLVRPPYSYSALIAMAIHGAPERRLTLSQIYQYVAD 162
UN_46_tnig      GPTGPGGPGGELSWFSLPSQEDLMKLVRPPYSYSALIAMAIHGAPDKRLTLSQIYQYVAD 164
FoxI3a_drer     GASGIGGSEGAFSWFSLPSQEDLMKLVRPPYSYSALIAMAIHGAPNRRVTLSQIYQYVAD 148
FoxI2_drer      SNS-SGFAGPDLGWLSIASQEELLKLVRPPYSYSALIAMAIQNAHEKKLTLSQIYQYVAD 161
FoxI1c_xlae     SNS-SSFCGTDLSWLSVASQEELLKVVRPPYSYSALIAMAIQNAPEKKLTLSQIYQYVAE 161
FoxI1_drer      LPPPTGFGSADLGWLSISSQQELFKMVRPPYSYSALIAMAIQNAQDKKLTLSQIYQYVAD 173
Foxi2_mmus      LGASGGLAGADLAWLSLSGQQELLRLVRPPYSYSALIAMAIQSAPLRRLTLSQIYQYVAG 149

FOXI1_hsap      NFPFYNKSKAGWQNSIRHNLSLNDCFKKVPRDEDDPGKGNYWTLDPNCEKMFDNGNFRRK 215
Foxi1_mmus      NFPFYNKSKAGWQNSIRHNLSLNDCFKKVPRDEDDPGKGNYWTLDPNCEKMFDNGNFRRK 209
FoxI1_xlae      NFPFYNKSKAGWQNSIRHNLSLNDCFKKVPRDEDDPGKGNYWTLDPNCEKMFDNGNFRRK 215
FoxI3b_drer     NFPFYNKSKAGWQNSIRHNLSLNDCFKKVPRDEDDPGKGNYWTLDPNCEKMFDNGNFRRK 222
UN_46_tnig      NFPFYNKSKAGWQNSIRHNLSLNDCFKKVPRDEDDPGKGNYWTLDPNCEKMFDNGNFRRK 224
FoxI3a_drer     NFPFYNKSKASWQNSIRHNLSLNDCFMKVPRDDSDPGKGNYWTLDPNCEKMFDNGNFRRK 208
FoxI2_drer      NFPFYKKSKAGWQNSIRHNLSLNDCFKKVPRDEDDPGKGNYWTLDPNCEKMFDNGNFRRK 221
FoxI1c_xlae     NFPFYKRSKAGWQNSIRHNLSLNDCFKKVPRDEDDPGKGNYWTLDPNCEKMFDNGNFRRK 221
FoxI1_drer      NFPFYKKSKAGWQNSIRHNLSLNDCFKKVARDEDDPGKGNYWTLDPNCEKMFDNGNFRRK 233
Foxi2_mmus      NFPFYKRSKAGWQNSIRHNLSLNDCFKKVPRDENDPGKGNYWTLDPNCEKMFDNGNFRRK 209

FOXI1_hsap      RKRKSDVSSS-TASLALEKTESSLPVDSPKTTEPQDILDGASPGGTTSSPEKRPSPPPSG 274
Foxi1_mmus      RKRKSDSSSS-TSSLASEKTENGLLASSPKPTEPQEVLDTASPDTTTSSPEKRSSPAPSG 268
FoxI1_xlae      RKRKSDVSP--NGQLSSDKPEGSPLSESPTNGEHQDMLGNSSPG-TDDSPEKR-SPPPSI 271
FoxI3b_drer     RKRKSDSLPE-KSSSGGNESGDSNGRGSPKSQ-SIDI---------STSPEKGPSPASTG 271
UN_46_tnig      RKRKSDTLPNGDGVSGGPESGD-NDRGSPKHNPALNI---------SPPTDRIPSPSSSG 274
FoxI3a_drer     RKRKSDSQAE---EEGKGYSGSDSALSSPKNP--------------SDSSERGNSPISTD 251
FoxI2_drer      RKRRSDSSTG-VSSNTKPEDDRQLAGIKPTDSPHLTGP-ASPDADAATDSHKGASPAGLA 279
FoxI1c_xlae     RKRRSDSSSA-EAVTVKGEEGRPALGGKGGESPSMLTP-SSPELEAASDDRKSTSPSGIT 279
FoxI1_drer      RKRRADGNAMSVKSEDALKLADTSSLMSASQPSLQNSP-------TSSDPKSSPSPSAEH 286
Foxi2_mmus      RRRRGE------TSEAAVPGAS-SPEGTALEP--RGS--------TPQDPQTSPSPSEAT 252
```

```
FOXI1_hsap       -APCLNSFLSSMTAYVSGG-SPTSHPLVTPGLSPEPSDKTGQNSLT--FNSFSPLTNLSN 330
Foxi1_mmus       -TPCLNNFLSTMTAYVSGT-NPISRSVATPGLSSEPIDKMGQNSLN--FNSYTPLTNLSS 324
FoxI1_xlae       -TPCLNNFLSSMTAYVNSA-TPISRSVP-LGLSNETSDKMGQNMVG--FNSYTPLSNMPS 326
FoxI3b_drer      PSPCLSNFLTEMSGVAAGSLDMEADPLSRPFTLSLPVDGAQRASQTTGFSTFTPSTTVSD 331
UN_46_tnig       VAPCLSSFLTEMSGVTGGAASEVGGDLSRP--LQLPVDDSHQPAQPPTFSSYSPSPGGSE 332
FoxI3a_drer      QAPCLNSFLNQMGDVASGSREAL---LPSP--LAVPL--SQRSSPTGVYGSYSPNATMPQ 304
FoxI2_drer       SAPCFNNFFNSMSALGSSS-TPTSRHGS----LGLVNELSSRNISA--LSPYHASTGPEA 332
FoxI1c_xlae      SSPCLNNFFSSMTSLDTTS---VNRQMS----LGLVNELSQRNITG--LGSF--TSGSIA 328
FoxI1_drer       -SPCFSNFIGNMNSIMSGN-AVRSRDGS----SAHLGDFTQHGMSG---HEISPPSEPGH 337
Foxi2_mmus       -TTCLSGFSTAMGALAGGF-GALP-DG-----LAH--DFSLR---------RPPPTAAAH 293

FOXI1_hsap       HSGGGDWANPMPTNM----LSYGG-SVLSQFS---PHFYNSVNTSGVLYPREGTEV 378
Foxi1_mmus       HGNGGEWANPVATNA----LGYGG-SVFNQFS---PHFYNSINTNGILFPREGTEV 372
FoxI1_xlae       HG-GSDWSSTVSSNP----FGYSS-SVFNQFT---PHFYNSMSTNNTLYNREGTEV 373
FoxI3b_drer      WASPLPPPPPMSSSPSHSTLAYSG-PVLSQFN---GHFFPGLSSTGILYPREGTEV 383
UN_46_tnig       WVPQVPGAPVLSTSPTPSSIGYAS-PILSQFTGSTGHFYPTLESAGVIYHREGTEV 387
FoxI3a_drer      WETQIPQSS-ISSTPYKD--GYSD-SMLNPYS---SQLYPVLGSSDLLYPREGSEV 353
FoxI2_drer       GGAPELQD-SVHVNRGMYYNSFTG-GQSTQFN---GHFYNSFSVNSLIYPRDGTEL 383
FoxI1c_xlae      EPSVDLQDNSLHLNRPSYYSTFSSTHQNNQFN---SHFYNTFSVNSLIYAREGSEV 381
FoxI1_drer       LNTNRLNYYSASHNN-------SG-----LIN----SISNHFSVNNLIYHRDGSEV 377
Foxi2_mmus       -SPQIPN--TAPGFA-------PG-----HQT----GATG-FRMGHLIYSRDGTEV 329
```

D. Alignment of the FoxO cluster of sequences.

Conserved and functionally important regions noted in the literature are highlighted. Green: forkhead domain (NCBI Protein database, see Additional file 1 for accession numbers) Orange: nuclear export signal [8-11] Black Box: nuclear localization signal [8, 9, 12] Blue: phosphorylation site [13-20] Grey Box: 14-3-3 binding site [8, 16, 17, 21, 22] Yellow: transactivation domain [23, 24] Red Box: EH1 motif The positively selected site identified by branch-site analysis is indicated by a purple box.

```
FoxO5_drer       MAETT-LEP-LSSLDIAIDPDFEPQKRPRSCTWPLP--ESSMAKPASNDTDIIPEEEDDE 56
FoxO5_xmac       MAEAP-LPDTLPDLDVVIDPDFEPQKRPRSCTWPLPRPDSSAVKPESTEADIIPEEEDDE 59
FOXO3a_hsap      MAEAPASPAPLSPLEVELDPEFEPQSRPRSCTWPLQRPELQAS-PAKPSGETAADSMIPE 59
Foxo3_mmus       MAEAPASPVPLSPLEVELDPEFEPQSRPRSCTWPLQRPELQAS-PAKPSGETAADSMIPE 59
UN_53_tnig       MAEAPRDEPPSN---VEIDPDFEPQKRPRSCTWPLPRPESGGK-PGTHDTDTPAAAALS- 55
Foxo6_mmus       ------MAAKLRAHQVDVDPDFAPQSRPRSCTWPLPQPDLAGDEDGALGAGVAEGSEDCG 54
FOXO4_hsap       ----MRIQPQKAAAIIDLDPDFEPQSRPRSCTWPLPRPEIANQPSEPPEVEPDLGEKVHT 56
Foxo4_mmus       MDPENKKSATGAAAILDLDPDFEPQSRPRSCTWPLPRPDLATEPHEPSEVEPSLGQKVPT 60
FOXO1a_hsap      --------MAEAPQVVEIDPDFEPLPRPRSCTWPLPRPEFSQSNSATSSPAPSGSAAANP 52
FoxO1a_sscr      --------MAEAPQVVEIDPDFEPLPRPRSCTWPLPRPEFSQSNSATSSPAPSGGPAANP 52
FoxO1a_stri      --------MAEAPQVVEIDPDFEPLPRPRSCTWPLPRPEFSQSNSATSSPAPSGGATANP 52
Foxo1_mmus       --------MAEAPQVVETDPDFEPLPRQRSCTWPLPRPEFNQSNSTTSSPAPSGGAAANP 52

FoxO5_drer       DD----SAMTINANGMGEGEDNG-------------------------SPSLAEELISIN 87
FoxO5_xmac       EDSATPTAITVNGSAAATEDQSS-------------------------NSPITDGAFPSP 94
FOXO3a_hsap      EEDDEDDEDGGGRAGSAMAIGGGGGSGTLGSGLLLED----SARVLAPGGQDPGSGPATA 115
Foxo3_mmus       EDDDEDDEDGGGRASSAMVIGGG-VSSTLGSGLLLED----SAMLLAPGGQDLGSGPASA 114
UN_53_tnig       ------------------------------------------------------------
Foxo6_mmus       PE---------------------------------------------------------- 56
FOXO4_hsap       EG---------------------------------------------------------- 58
Foxo4_mmus       EG---------------------------------------------------------- 62
FOXO1a_hsap      DAAAGLPSASAAAVSADFMSNLSLLEESEDFPQAPG------SVAAAVAAAAAAAATGGL 106
FoxO1a_sscr      DAAAGLPSASAAAVNADFMSNLSLLEESGDFQQAPGSVAAAAAAAAAVAAAAAAAATGGL 112
FoxO1a_stri      DASAGLPPASAAAVSADFMSNLSLLEESEDFPQAPG-------SVAAAVAAAAAAATGGL 105
Foxo1_mmus       DAAASL--ASASAVSTDFMSNLSLLEESEDFARAPG----------CVAVAAAAAASRGL 100

FoxO5_drer       GQENTGSPLSSQAASATT--ASPEVASQQQ---------------TPRK-SSSRRNAWGN 129
FoxO5_xmac       GHDSGGSPLSTHSPTATSGALTPSGLPAAQ---------------TPRK-ASSRRNAWGN 138
FOXO3a_hsap      AGGLSGGTQALLQPQQPLPPPQPGAAGGSG---------------QPRK-CSSRRNAWGN 159
Foxo3_mmus       AGALSGGTPTQLQPQQPLPQPQPGAAGGSG---------------QPRK-CSSRRNAWGN 158
UN_53_tnig       -------------------------ASASQ---------------QLRK-SSARRNAWGN 74
Foxo6_mmus       ----------RRATAPAMAPAPPLGAEVGP---------------LRKA-KSSRRNAWGN 90
FOXO4_hsap       ---RSEPILLPSRLSEPAGGPQPGILGAVT---------------GPRK-GGSRRNAWGN 99
Foxo4_mmus       ---HSEPILLPSRLPEPAGGPQPGILGAVT---------------GPRK-GGSRRNAWGN 103
FOXO1a_hsap      CGDFQGPEAGCLHPAPPQ-PPPPGPLSQHPPVPP---AAAGPLAGQPRKSSSSRRNAWGN 162
FoxO1a_sscr      CGDFQGPEAGCLHPAPPQQPPPPGPLSQHPPVPP---AAAGSLAGQPRKSSSSRRNAWGN 169
FoxO1a_stri      CGDFQGLEAGCLHPAPPQ-PPPPGPLSQHPPVPP---AAG-PLAGQPRKSSSSRRNAWGN 160
Foxo1_mmus       CGDFQGPEAGCVHPAPPQ-PPPTGPLSQPPPVPPSAAAAAGPLAGQPRKTSSSRRNAWGN 159

FoxO5_drer       LSYADLITKAIESTPDKRLTLSQIYDWMVSSVPYFKDKGDSNSSAGWKNSIRHNLSLHSR 189
FoxO5_xmac       LSYADLITKAIESSPEKRLTLSQIYDWMVRSIPYFKDKGDSNSSAGWKNSIRHNLSLHSR 198
FOXO3a_hsap      LSYADLITRAIESSPDKRLTLSQIYEWMVRCVPYFKDKGDSNSSAGWKNSIRHNLSLHSR 219
Foxo3_mmus       LSYADLITRAIESSPDKRLTLSQIYEWMVRCVPYFKDKGDSNSSAGWKNSIRHNLSLHSR 218
UN_53_tnig       YSYADLITQAIESSPEKRLTLSQIYDWMVRSVPYFKDKGDSNSSAGWKNSIRHNLSLHSR 134
Foxo6_mmus       LSYADLITKAIESAPDKRLTLSQIYDWMVRYVPYFKDKGDSNSSAGWKNSIRHNLSLHTR 150
FOXO4_hsap       QSYAEFISQAIESAPEKRLTLAQIYEWMVRTVPYFKDKGDSNSSAGWKNSIRHNLSLHSK 159
Foxo4_mmus       QSYAELISQAIESAPEKRLTLAQIYEWMVRTVPYFKDKGDSNSSAGWKNSIRHNLSLHSK 163
FOXO1a_hsap      LSYADLITKAIESSAEKRLTLSQIYEWMVKSVPYFKDKGDSNSSAGWKNSIRHNLSLHSK 222
FoxO1a_sscr      LSYADLITKAIESSAEKRLTLSQIYEWMVKSVPYFKDKGDSNSSAGWKNSIRHNLSLHSK 229
FoxO1a_stri      LSYADLITKAIESSAEKRLTLSQIYEWMVKSVPYFKDKGDSNSSAGWKNSIRHNLSLHSK 220
Foxo1_mmus       LSYADLITKAIESSAEKRLTLSQIYEWMVKSVPYFKDKGDSNSSAGWKNSIRHNLSLHSK 219
```

```
FoxO5_drer      FIRVQNEGTGKSSWWMINPEGGRGGKAPRRRAVSMDNGNKYTKTARGR-AAKKKAALQAA 248
FoxO5_xmac      FIRVQNEGTGKSSWWMINPEGGKGGKAPRRRAVSMDNSNKYTKSARGRAAAKKKAALQAV 258
FOXO3a_hsap     FMRVQNEGTGKSSWWIINPDGGKSGKAPRRRAVSMDNSNKYTK-SRGR-AAKKKAALQTA 277
Foxo3_mmus      FMRVQNEGTGKSSWWIINPDGGKSGKAPRRRAVSMDNSNKYTK-SRGR-AAKKKAALQAA 276
UN_53_tnig      FVKVQNEGTGKSSWWMVNPEGGKGGKAPRRRAVSMDNSKYIKG-ARGR-ATKKKALLQAA 192
Foxo6_mmus      FIRVQNEGTGKSSWWMLNPEGGKTGKTPRRRAVSMDNGAKFLR-IKGKASKKKQLHLPER 209
FOXO4_hsap      FIKVHNEATGKSSWWMLNPEGGKSGKAPRRRAASMDSSSKLLR-GRSKAPKKKPSVLPAP 218
Foxo4_mmus      FIKVHNEATGKSSWWMLNPDGGKGGKAPRRRAASMDSSSKLLR-GRSKGPKKKPSVLPAP 222
FOXO1a_hsap     FIRVQNEGTGKSSWWMLNPEGGKSGKSPRRRAASMDNNSKFAK-SRSR-AAKKKASLQSG 280
FoxO1a_sscr     FIRVQNEGTGKSSWWMLNPEGGKSGKSPRRRAASMDNNSKFAK-SRGR-AAKKKASLQSG 287
FoxO1a_stri     FIRVQNEGTGKSSWWMLNPEGGKSGKSPRRRAASMDNNSKFAK-SRGQ-AAKKKASLQSG 278
Foxo1_mmus      FIRVQNEGTGKSSWWMLNPEGGKSGKSPRRRAASMDNNSKFAK-SRGR-AAKKKASLQSG 277

FoxO5_drer      QEGSLEN--ISAGGLS---KWPGSPTSRSSDEQESCWTDFRSRTNSNASTVSGRLSPILA 303
FoxO5_xmac      AAAAGEGGGDSPTGPS---KWPGSPTSRSSEE-LDAWTDFRSRTNSNASTLSGRLSPILA 314
FOXO3a_hsap     PESADDS----PSQLS---KWPGSPTSRSSDE-LDAWTDFRSRTNSNASTVSGRLSPIMA 329
Foxo3_mmus      PESADDS----PSQLS---KWPGSPTSRSSDE-LDAWTDFRSRTNSNASTVSGRLSPILA 328
UN_53_tnig      QDGSSES----SSSLS---KWTGSPTSRSSDE-LDAWTDFRSRTNSNASTLSGRLSPILA 244
Foxo6_mmus      SPDDSPPGAPVPGPLSASAKWAASPASHASDD-YEAWADFRGSRRP--------LLGEAA 260
FOXO4_hsap      PEGATPT-----SPVGHFAKWSGSPCSRNREE-ADMWTTFRPRSSSNASSVSTRLSPLRP 272
Foxo4_mmus      PEGATPR-----SPLGHFAKWSSSPCPRNREE-ADVWTTFRPRSSSNASTVSTRLSPMRP 276
FOXO1a_hsap     QEGAGDS------PGSQFSKWPASPGSHSNDD-FDNWSTFRPRTSSNASTISGRLSPIMT 333
FoxO1a_sscr     QEGAGDS------PGSQFSKWPASPGSHSNDD-FDNWSTFRPRTSSNASTISGRLSPIMT 340
FoxO1a_stri     QEGAGDS------PGSQFSKWPASPGSHSNDD-FDNWSTFRPRTSSNASTISGRFSPIMT 331
Foxo1_mmus      QEGPGDS------PGSQFSKWPASPGSHSNDD-FDNWSTFRPRTSSNASTISGRLSPIMT 330

FoxO5_drer      NPELDDVSDG-PPLSPILYSSPSSLSPPSSTSSNSKTCPGELPVLADLTGTMNLNE--AD 360
FoxO5_xmac      NPELDEVADDEPPLSPMIYSSPGRALSPGNT--NGKAVPTELPRLADLANTMNLNDGITQ 372
FOXO3a_hsap     STELDEVQDDDAPLSPMLYSSSASLSP-----SVSKPCTVELPRLTDMAGTMNLNDGLTE 384
Foxo3_mmus      STELDDVQDDDGPLSPMLYSSSASLSP-----SVSKPCTVELPRLTDMAGTMNLNDGLAE 383
UN_53_tnig      NLELDEVPDDDSPLSPMLYSSPSSMSP-----STGP------TVLSDLAGTMNLNDGLSD 293
Foxo6_mmus      ELEDDEALEALAPSSPLMYPSPASALSP----ALGARCPGELPRLAELGGPLGLHGGGVA 316
FOXO4_hsap      ESEVLAEEIP-ASVSSYAGGVP-----------------------PTLNEGLELLDGLNL 308
Foxo4_mmus      ESEVLAEEEMPASASSYAGGVP-----------------------PTLSEDLELLDGLNL 313
FOXO1a_hsap     EQDDLGEGDVHSMVYPPSAAKMASTLP----------------SLSEISNPENMEN--LL 375
FoxO1a_sscr     EQDDLGNGDVHSMVYPPSAAKMASTLP----------------SLSEISNPENMEN--LL 382
FoxO1a_stri     EQDDLGDGDVHSLVYPPSASKMASTLP----------------SLSEISNPENMEN--LL 373
Foxo1_mmus      EQDDLGDGDVHSLVYPPSAAKMASTLP----------------SLSEISNPENMEN--LL 372

FoxO5_drer      NLMVDLLDNI----ATQVQVVQE-SSPFSFGSK--STASPPSTSTSQTVSNNTGG---FS 410
FoxO5_xmac      DLMDDFLDNIKLVPTTCSQAMQNGSSGFSFGSKPNGIGSPSSTSSPSSNNSSNGGGNSYS 432
FOXO3a_hsap     NLMDDLLDNITLPPSQPSPTGGLMQRSSSFPYTTKGSGLGSPTSS-------------FN 431
Foxo3_mmus      NLMDDLLDNIALPPSQPSPPGGLMQRGSSFPYTAKSSGLGSPTGS-------------FN 430
UN_53_tnig      NLMDDLLDNISLTATQQLPPG----EEDSGGQATSVFTFSCSGSS-------------LG 336
Foxo6_mmus      GLPDALLDGAQDAYGPRARAG----TPSYFGSCKASAYG-------------------G 352
FOXO4_hsap      TSSHSLLSRSGLSGFSLQHPG--------------------------------------- 329
Foxo4_mmus      ASPHSLLSRSGLSGFSLQHPG--------------------------------------- 334
FOXO1a_hsap     DNLNLLSSPTSLTVSTQSSPGTMMQQTPCYSFAPPNTSLNSPSPN-------------YQ 422
FoxO1a_sscr     DNLNLLSSPTSLTVSTQSSPGTIMQQTPCYSFAPPNTSLNSPSPN-------------YQ 429
FoxO1a_stri     DNLNLLSSPTSLTVSTQSSPGSMMQQTPCYSFAPPSTSLNSPSPN-------------YQ 420
Foxo1_mmus      DNLNLLSSPTSLTVSTQSSPGSMMQQTPCYSFAPPNTSLNSPSPN-------------YS 419
```

```
FoxO5_drer      NPIFGPSPG--SLRQKPMQTIQENKQTSFSSISCFETSLQDLLNSDSLVSHSH-SDVMMT 467
FoxO5_xmac      NSIFSPYTAGSSLRPSPMQTIQENKQTSFSTTGMSHFGSHTLQDLLNSDSHNH-SDVMMT 491
FOXO3a_hsap     STVFGPSSLN-SLRQSPMQTIQENKPATFSSMSHYG--NQTLQDLLTSDSLSH-SDVMMT 487
Foxo3_mmus      STVFGPSSLN-SLRQSPMQTIQENRPATFSSVSHYG--NQTLQDLLASDSLSH-SDVMMT 486
UN_53_tnig      SPLFSPPSIT-SLRQSPMQTIQENKQTTFSCVSHFGD-HQSLQDLLSLDSHGH-SNVMLT 393
Foxo6_mmus      GGGFGPPALG-SLRRLPMQTIQENKQASFVQAAAPFR-PGALPALLPPPPPAP-RPGPLL 409
FOXO4_hsap      --VTGPLHTYSSSLFSPAEGPLSAGEGCFSSS-------QALEALLTSDTPPPPADVLMT 380
Foxo4_mmus      --LAGPLHSYGASLFGPIDGSLSAGEGCFSSS-------QSLEALLTSDTPPPPADVLMT 385
FOXO1a_hsap     KYTYGQSSMS-PLPQMPIQTLQDNK-SSYGGMSQYNCAPGLLKELLTSDSPPH--NDIMT 478
FoxO1a_sscr     KYTYGQSSMS-PLPQMPMQTLQDSK-SSYGGMAQYNCAAGLLKELLTSDSPPH--NDIMT 485
FoxO1a_stri     KYTYGQSSMS-PLSQMPMQTLQDNK-SSYGGLNQFNCAQGLLKELLTSDSPPH--NDIMS 476
Foxo1_mmus      KYTYGQSSMS-PLPQMPMQTLQDSK-SSYGGLNQYNCAPGLLKELLTSDSPPH--NDIMS 475


FoxO5_drer      QSDPLISQASAAVTSQNYRLRNNPMLRNDPMMSSFSSVLNGRSGHLQNK-QHHQGSNGQG 526
FoxO5_xmac      QSDPLMSQASAVAIIS-QNSRRNVMVRNDPVMT-FGTTGGLQSSHRETL-QTN--NHNQS 546
FOXO3a_hsap     QSDPLMSQASTAVSAQ--NSRRNVMLRNDPMMSFAAQPNQGSLVNQNLLHHQHQTQGALG 545
Foxo3_mmus      QSDPLMSQASTAVSAQ--NARRNVMLRNDPMMSFAAQPTQGSLVNQNLLHHQHQTQGALG 544
UN_53_tnig      QSDPLMSQASTAVALQ--NSRRNAMLLHKDPPSVNHTGAG-----------QAQSSSVPG 440
Foxo6_mmus      GAPGELALAGAAAAYP--GKGAAPYAPPAPSRSALAHPIS--------------LMTLPG 453
FOXO4_hsap      QVDPILSQAPTLLLLG-----------GLPSSSKLATGVG----------------LCPK 413
Foxo4_mmus      QVDPILSQAPTLLLLG-----------GMPSSSKLGTGVS----------------LCPT 418
FOXO1a_hsap     PVDPGVAQPNSRVLGQNVMMGPNSVMSTYGSQASHNKMMNPSSHTHPGHAQQTSAVNGRP 538
FoxO1a_sscr     PVDPGVAQPNSRVLGQNVLMGPSSVMPAYGGQASHNKMMNPSSHSHPGHGQSTSAVNGRA 545
FoxO1a_stri     SVDPGVAQPNSRVLGQNVMLGPNSVMPAYGNQASHNKMMNPSSHTHPGHAQQTSVVNGRA 536
Foxo1_mmus      PVDPGVAQPNSRVLGQNVMMGPNSVMPAYGSQASHNKMMNPSSHTHPGHAQQTASVNGRT 535


FoxO5_drer      SLRSLSNNRAQSLVNDANNLVSAKQLLQPQPT-MLMTETPLYSGMNGSNGLGICHPSGTD 585
FoxO5_xmac      TVRSLNG--DLNLANEANTLANVKQQLLLSPIGGNGTSSMQIDTSIFLNGTASSSGGCQD 604
FOXO3a_hsap     GSRALSNSVSNMGLSESSSLGSAKHQQQSPVSQSMQTLSDSLSGSSLYSTSANLPVMGHE 605
Foxo3_mmus      GSRALSNSVSNMGLSDSSSLGSAKHQQQSPASQSMQTLSDSLSGSSLYSASANLPVMGHD 604
UN_53_tnig      WQAGLSTSEDNGGRNNAKQP----HLKSPSKNASMQLGSGFPS---------------QD 481
Foxo6_mmus      EAGAAGLAPPAHAAAFGGPPGGLLLDALPGPYAAAAGPLGAG----------------PD 498
FOXO4_hsap      PLEARGPSSLVPTLSMIAPPPVMASAPIPKALGTPVLTPPTEAAS-------------QD 460
Foxo4_mmus      PLEGPGPSNLVPNLSVMAPPPVMAGAPIPKVLGTPVLASPTEDSS-------------HD 465
FOXO1a_hsap     LPHTVSTMPHTSGMNRLTQVKTPVQVPLPHPMQMSALGGYSSVSSCN--GYGRMGLLHQE 596
FoxO1a_sscr     LPHAVNTMPHASGMNRLAQEKTALQVPLPHPMQMNALGGYSPASTCN--GYGRMGLLHQE 603
FoxO1a_stri     LPHTVNTMPHTSGMNRLTPVKTPLQVPLLHHMQMSALGGYSSVSSCS--GYGRMGVLHQE 594
Foxo1_mmus      LPHVVNTMPHTSAMNRLTPVKTPLQVPLSHPMQMSALGSYSSVSSCN--GYGRMGVLHQE 593


FoxO5_drer      RFPSDLDLDIFN-GSLDCDVDSIIRSELMDSDGLDFNFDALMQNA-VSLNPVGNFTGTKQ 643
FoxO5_xmac      RFPTDLDLDMFNSGSLDCDMESIIRNDLMDADGLDFNFESLAN-----MNGVSNFTSTK- 658
FOXO3a_hsap     KFPSDLDLDMFN-GSLECDMESIIRSELMDADGLDFNFDSLISTQNVVGLNVGNFTGAKQ 664
Foxo3_mmus      KFPSDLDLDMFN-GSLECDMESIIRSELMDADGLDFNFDSLISTQNVVGLNVGNFTGAKQ 663
UN_53_tnig      RFPADLDLDVFN-SSLECDMDAIIRNELMDADCLDLSFDSRLTPTQNGNKNSGSYSGSKP 540
Foxo6_mmus      RFPADLDLDMFS-GSLECDVESIILNDFMDSDEMDFNFDSALP------PPPPGLAGAPP 551
FOXO4_hsap      RMPQDLDLDMYM-ENLECDMDNIISDLMDEGEGLDFNFEPDP------------------ 501
Foxo4_mmus      RMPQDLDLDMYM-ENLECDMDNIISDLMD-GEGLDFNFEPDP------------------ 505
FOXO1a_hsap     KLPSDLDGMFIE--RLDCDMESIIRNDLMDGDTLDFNFDNVLP--------NQSFPHSVK 646
FoxO1a_sscr     KLPSDLDGMFIE--RLDCDMESIIRNDLMDGDTLDFNFDNVLP--------NQSFPHSVK 653
FoxO1a_stri     KLPSDLDGMFIE--RLDCDMESIIRNDLMDGDALDFNFDNVLP--------NQSFPHGVK 644
Foxo1_mmus      KLPSDLDGMFIE--RLDCDMESIIRNDLMDGDTLDFNFDNVLP--------NQSFPHSVK 643
```

```
FoxO5_drer       SN-HSWVPG 651
FoxO5_xmac       ---HSWVPG 664
FOXO3a_hsap      ASSQSWVPG 673
Foxo3_mmus       ASSQSWVPG 672
UN_53_tnig       AAPRSWVPS 549
Foxo6_mmus       PN-QSWVPG 559
FOXO4_hsap       ---------
Foxo4_mmus       ---------
FOXO1a_hsap      TTTHSWVSG 655
FoxO1a_sscr      TTTHSWVSG 662
FoxO1a_stri      TTTHSWVSG 653
Foxo1_mmus       TTTHSWVSG 652
```

E. Alignment of the FoxP cluster of sequences.

Conserved and functionally important regions noted in the literature are highlighted. Green: forkhead domain (NCBI Protein database, see Additional file 1 for accession numbers) Purple: glutamine rich region [25-29] Blue: zinc finger, sites 288-311 [25, 28-30] Yellow: leucine zipper, sites 324-349 [25, 29-31] Red Box: EH1 motif

```
FOXP1_hsap      MMQESGTETKSNGSAIQNGSGGSNHLLE-----------CGGLREGRSNGETPAVDIGAA 49
Foxp1_mmus      MMQESGSETKSNGSAIQNGSSGGNHLLE-----------CGALRDTRSNGEAPAVDLGAA 49
FoxP2_ggor      MMQESATETISNSSMNQNGMSTLSSQLD-----------AGS-RDGRSSGDT-SSEVSTV 47
FoxP2_mmul      MMQESATETISNSSMNQNGMSTLSSQLD-----------AGS-RDGRSSGDT-SSEVSTV 47
Foxp2_mmus      MMQESATETISNSSMNQNGMSTLSSQLD-----------AGS-RDGRSSGDT-SSEVSTV 47
FoxP2_ppyg      MMQESVTETISNSSMNQNGMSTLSSQLD-----------AGS-RDGRSSGDT-SSEVSTV 47
FoxP2_ptro      MMQESATETISNSSMNQNGMSTLSSQLD-----------AGS-RDGRSSGDT-SSEVSTV 47
FOXP2_hsap      MMQESATETISNSSMNQNGMSTLSSQLD-----------AGS-RDGRSSGDT-SSEVSTV 47
FoxP2_tgut      MMQESATETISNSSMNQNGMSTLSSQLD-----------AGS-RDGRSSGDT-STEVSTV 47
FOXP4_hsap      MMVESASETIRSAPSGQNGVGSLSGQADGSSGGATGTTASGTGREVTTGADS-NGEMSPA 59

FOXP1_hsap      DLAHAQQQQQQALQVARQLLLQQQQQQQ------------------------------VS 79
Foxp1_mmus      DLAHVQQQQQQALQVARQLLLQQQQQQQQQQQQQQQQQQQQQQQQQQQQQQQQQQQQQVS 109
FoxP2_ggor      ELLHLQQQQ--ALQAARQLLLQQQT----------------------------------S 71
FoxP2_mmul      ELLHLQQQQ--ALQAARQLLLQQQT----------------------------------S 71
Foxp2_mmus      ELLHLQQQQ--ALQAARQLLLQQQT----------------------------------S 71
FoxP2_ppyg      ELLHLQQQQ--ALQAARQLLLQQQT----------------------------------S 71
FoxP2_ptro      ELLHLQQQQ--ALQAARQLLLQQQT----------------------------------S 71
FOXP2_hsap      ELLHLQQQQ--ALQAARQLLLQQQT----------------------------------S 71
FoxP2_tgut      ELLHLQQQQ--ALQAARQLLLQQQT----------------------------------S 71
FOXP4_hsap      ELLHFQQQQ--ALQVARQFLLQQAS----------------------------------- 82

FOXP1_hsap      GLKSPKRND-KQ--PALQVPVSVAMMTPQVITPQQMQQILQQQVLSPQQLQVLLQQQQAL 136
Foxp1_mmus      GLKSPKRND-KQ--PALQVPVSVAMMTPQVITPQQMQQILQQQVLSPQQLQVLLQQQQAL 166
FoxP2_ggor      GLKSPKSSD-KQ--RPLQVPVSVAMMTPQVITPQQMQQILQQQVLSPQQLQALLQQQQAV 128
FoxP2_mmul      GLKSPKSSD-KQ--RPLQVPVSVAMMTPQVITPQQMQQILQQQVLSPQQLQALLQQQQAV 128
Foxp2_mmus      GLKSPKSSE-KQ--RPLQVPVSVAMMTPQVITPQQMQQILQQQVLSPQQLQALLQQQQAV 128
FoxP2_ppyg      GLKSPKSSD-KQ--RPLQVPVSVAMMTPQVITPQQMQQILQQQVLSPQQLQALLQQQQAV 128
FoxP2_ptro      GLKSPKSSD-KQ--RPLQVPVSVAMMTPQVITPQQMQQILQQQVLSPQQLQALLQQQQAV 128
FOXP2_hsap      GLKSPKSSD-KQ--RPLQVPVSVAMMTPQVITPQQMQQILQQQVLSPQQLQALLQQQQAV 128
FoxP2_tgut      GLKSPKGSE-KQ--RPLQVPVSVAMMTPQVITPQQMQQILQQQVLSPQQLQALLQQQQAV 128
FOXP4_hsap      GLSSPGNNDSKQSASAVQVPVSVAMMSPQMLTPQQMQQIL-----SPPQLQALLQQQQAL 137

FOXP1_hsap      MLQQQQLQEFYKKQQEQLQLQLLQQQ---------------------------------- 162
Foxp1_mmus      MLQQQ-LQEFYKKQQEQLQLQLLQQQ---------------------------------- 191
FoxP2_ggor      MLQQQQLQEFYKKQQEQLHLQLLQQQQQQQQQQQQQQQQQQQQQQQQQQQQQQQQQQQQQ 188
FoxP2_mmul      MLQQQQLQEFYKKQQEQLHLQLLQQQQQQQQQQQQQQQQQQQQQQQQQQQQQQQQQQQQQ 188
Foxp2_mmus      MLQQQQLQEFYKKQQEQLHLQLLQQQQQQQQQQQQQQQQQQQQQQQQQQQQQQQQQQQQQ 188
FoxP2_ppyg      MLQQQQLQEFYKKQQEQLHLQLLQQQQQQQQQQQQQQQQQQQQQQQQQQQQQQQQQQQQQ 188
FoxP2_ptro      MLQQQQLQEFYKKQQEQLHLQLLQQQQQQQQQQQQQQQQQQQQQQQQQQQQQQQQQQQQQ 188
FOXP2_hsap      MLQQQQLQEFYKKQQEQLHLQLLQQQQQQQQQQQQQQQQQQQQQQQQQQQQQQQQQQQQQ 188
FoxP2_tgut      MLQQQQLQEFYKKQQEQLHLQLLQQQQQQQQQQQQQQQQQQQQQQQQQQQQQQQQQQQQQ 188
FOXP4_hsap      MLQQ--LQEYYKKQQEQLHLQLLTQQ---------------------------------- 161

FOXP1_hsap      ----HAGKQPKEQQQ-------VATQQLAFQQQLLQMQQLQQQ-HLLSLQRQGLLTIQPG 210
Foxp1_mmus      ----HAGKQPKEQQ--------VATQQLAFQQQLLQMQQLQQQ-HLLSLQRQGLLTIQPG 238
FoxP2_ggor      Q---HPGKQAKEQQQQQQQQQQLAAQQLVFQQQLLQMQQLQQQQHLLSLQRQGLISIPPG 245
FoxP2_mmul      QQ--HPGKQAKEQQQQQQQQQQLAAQQLVFQQQLLQMQQLQQQQHLLSLQRQGLISIPPG 246
Foxp2_mmus      QQQ-HPGKQAKEQQQQQQQQQ-LAAQQLVFQQQLLQMQQLQQQQHLLSLQRQGLISIPPG 246
FoxP2_ppyg      QQ--HPGKQAKEQQQQQQQQQ-LAAQQLVFQQQLLQMQQLQQQQHLLSLQRQGLISIPPG 245
FoxP2_ptro      QQQQHPGKQAKEQQQQQQQQQQLAAQQLVFQQQLLQMQQLQQQQHLLSLQRQGLISIPPG 248
FOXP2_hsap      QQQ-HPGKQAKEQQQQQQQQQQLAAQQLVFQQQLLQMQQLQQQQHLLSLQRQGLISIPPG 247
FoxP2_tgut      Q---HPGKQAKEQQQQQQ----LAAQQLVFQQQLLQMQQLQQQQHLLNLQRQGLISIPPG 241
FOXP4_hsap      ----QAGK-PQPKEA-------LGNKQLAFQQQLLQMQQLQQQ-HLLNLQRQGLVSLQPN 208
```

```
FOXP1_hsap      QPALPLQPLAQ-GMIPTELQQLWK-EVTSAHTAEETTGNNHSSLDLTT---TCVSSSAPS 265
Foxp1_mmus      QPALPLQPLAQ-GMIPTELQQLWK-EVTSAHTAEETTSSNHSSLDLTS---TCVSSSAPS 293
FoxP2_ggor      QAALPVQSLPQAGLSPAEIQQLWK-EVTGVHSMEDN-GIKHGGLDLTTNNSSSTTSSTTS 303
FoxP2_mmul      QAALPVQSLPQAGLSPAEIQQLWK-EVTGVHSMEDN-GIKHGGLDLTTNNSSSTTSSTTS 304
Foxp2_mmus      QAALPVQSLPQAGLSPAEIQQLWK-EVTGVHSMEDN-GIKHGGLDLTTNNSSSTTSSTTS 304
FoxP2_ppyg      QAALPVQSLPQAGLSPAEIQQLWK-EVTGVHSMEDN-GIKHGGLDLTTNNSSSTTSSTTS 303
FoxP2_ptro      QAALPVQSLPQAGLSPAEIQQLWK-EVTGVHSMEDN-GIKHGGLDLTTNNSSSTTSSTTS 306
FOXP2_hsap      QAALPVQSLPQAGLSPAEIQQLWK-EVTGVHSMEDN-GIKHGGLDLTTNNSSSTTSSNTS 305
FoxP2_tgut      QSALPVQSLPQAGLSPAEIQQLWK-EVTGVHSMEDN-GIKHGGLDLTTNNSSSTTSSTTS 299
FOXP4_hsap      QASGPLQTLPQ-AVCPTDLPQLWKGEGAPGQPAEDS--VKQEGLDLTGTAATATSFAAPP 265

FOXP1_hsap      KTSLIMNPHASTNGQLSVHTPKRESLSHEEHPHSHPLYGHGVCKWPGCEAVCEDFQSFLK 325
Foxp1_mmus      KSSLIMNPHASTNGQLSVHTPKRESLSHEEHPHSHPLYGHGVCKWPGCEAVCDDFPAFLK 353
FoxP2_ggor      KASPPITHHSIVNGQSSVLNARRDSSSHEETGASHTLYGHGVCKWPGCESICEDFGQFLK 363
FoxP2_mmul      KASPPITHHSIVNGQSSVLNARRDSSSHEETGASHTLYGHGVCKWPGCESICEDFGQFLK 364
Foxp2_mmus      KASPPITHHSIVNGQSSVLNARRDSSSHEETGASHTLYGHGVCKWPGCESICEDFGQFLK 364
FoxP2_ppyg      KASPPITHHSIVNGQSSVLNARRDSSSHEETGASHTLYGHGVCKWPGCESICEDFGQFLK 363
FoxP2_ptro      KASPPITHHSIVNGQSSVLNARRDSSSHEETGASHTLYGHGVCKWPGCESICEDFGQFLK 366
FOXP2_hsap      KASPPITHHSIVNGQSSVLSARRDSSSHEETGASHTLYGHGVCKWPGCESICEDFGQFLK 365
FoxP2_tgut      KASPPITHHSIVNGQSSVLNARRDSSSHEETGASHTLYGHGVCKWPGCESVCEDFGQFLK 359
FOXP4_hsap      KVSPPLSHHTLPNGQPTVLTSRRDSSSHEETPGSHPLYGHGECKWPGCETLCEDLGQFIK 325

FOXP1_hsap      HLNSEHALDDRSTAQCRVQMQVVQQLELQLAKDKERLQAMMTHLHVKSTEPKAAPQPLNL 385
Foxp1_mmus      HLNSEHALDDRSTAQCRVQMQVVQQLELQLAKDKERLQAMMTHLHVKSTEPKAAPQPLNL 413
FoxP2_ggor      HLNNEHALDDRSTAQCRVQMQVVQQLEIQLSKERERLQAMMTHLHMRPSEPKPSPKPLNL 423
FoxP2_mmul      HLNNEHALDDRSTAQCRVQMQVVQQLEIQLSKERERLQAMMTHLHMRPSEPKPSPKPLNL 424
Foxp2_mmus      HLNNEHALDDRSTAQCRVQMQVVQQLEIQLSKERERLQAMMTHLHMRPSEPKPSPKPLNL 424
FoxP2_ppyg      HLNNEHALDDRSTAQCRVQMQVVQQLEIQLSKERERLQAMMTHLHMRPSEPKPSPKPLNL 423
FoxP2_ptro      HLNNEHALDDRSTAQCRVQMQVVQQLEIQLSKERERLQAMMTHLHMRPSEPKPSPKPLNL 426
FOXP2_hsap      HLNNEHALDDRSTAQCRVQMQVVQQLEIQLSKERERLQAMMTHLHMRPSEPKPSPKPLNL 425
FoxP2_tgut      HLNNEHALDDRSTAQCRVQMQVVQQLEIQLSKERERLQAMMTHLHMRPSEPKPSPKPLNL 419
FOXP4_hsap      HLNTEHALDDRSTAQCRVQMQVVQQLEIQLAKESERLQAMMAHLHMRPSEPKPFSQP--- 382

FOXP1_hsap      VSSVTLSKSASEASPQSLPHTPTTPTAPLTPVTQGPSVITTTSMHTVGPIRRRYSDKYNV 445
Foxp1_mmus      VSSVTLSKSASEASPQSLPHTPTTPTAPLTPVTQGPSVITTTSMHTVGPIRRRYSDKYNV 473
FoxP2_ggor      VSSVTMSKNMLETSPQSLPQTPTTPTAPVTPITQGPSVITPASVPNVGAIRRRHSDKYNI 483
FoxP2_mmul      VSSVTMSKNMLETSPQSLPQTPTTPTAPVTPITQGPSVITPASVPNVGAIRRRHSDKYNI 484
Foxp2_mmus      VSSVTMSKNMLETSPQSLPQTPTTPTAPVTPITQGPSVITPASVPNVGAIRRRHSDKYNI 484
FoxP2_ppyg      VSSVTMSKNMLETSPQSLPQTPTTPTAPVTPITQGPSVITPASVPNVGAIRRRHSDKYNI 483
FoxP2_ptro      VSSVTMSKNMLETSPQSLPQTPTTPTAPVTPITQGPSVITPASVPNVGAIRRRHSDKYNI 486
FOXP2_hsap      VSSVTMSKNMLETSPQSLPQTPTTPTAPVTPITQGPSVITPASVPNVGAIRRRHSDKYNI 485
FoxP2_tgut      VSSVTMSKNMLETSPQSLPQTPTTPTAPVTPITQGPSVITPASVPNVGAIRRRHSDKYNI 479
FOXP4_hsap      ---VTVS--AADSFPDGLVHPPTSAAAPVTPLR--PPGLGSASLHGGGPARRRSSDKFCS 435

FOXP1_hsap      PISSADIAQNQEFYKNAEVRPPFTYASLIRQAILESPEKQLTLNEIYNWFTRMFAYFRRN 505
Foxp1_mmus      PISSADIAQNQEFYKNAEVRPPFTYASLIRQAILESPEKQLTLNEIYNWFTRMFAYFRRN 533
FoxP2_ggor      PMSS-EIAPNYEFYKNADVRPPFTYATLIRQAIMESSDRQLTLNEIYSWFTRTFAYFRRN 542
FoxP2_mmul      PMSS-EIAPNYEFYKNADVRPPFTYATLIRQAIMESSDRQLTLNEIYSWFTRTFAYFRRN 543
Foxp2_mmus      PMSS-EIAPNYEFYKNADVRPPFTYATLIRQAIMESSDRQLTLNEIYSWFTRTFAYFRRN 543
FoxP2_ppyg      PMSS-EIAPNYEFYKNADVRPPFTYATLIRQAIMESSDRQLTLNEIYSWFTRTFAYFRRN 542
FoxP2_ptro      PMSS-EIAPNYEFYKNADVRPPFTYATLIRQAIMESSDRQLTLNEIYSWFTRTFAYFRRN 545
FOXP2_hsap      PMSS-EIAPNYEFYKNADVRPPFTYATLIRQAIMESSDRQLTLNEIYSWFTRTFAYFRRN 544
FoxP2_tgut      PMSS-EIAPNYEFYKNADVRPPFTYATLIRQAIMESSDRQLTLNEIYSWFTRTFAYFRRN 538
FOXP4_hsap      PISS-ELAQNHEFYKNADVRPPFTYASLIRQAILETPDRQLTLNEIYNWFTRMFAYFRRN 494
```

```
FOXP1_hsap      AATWKNAVRHNLSLHKCFVRVENVKGAVWTVDEVEFQKRRPQKISGNPSLIKNMQSSHAY 565
Foxp1_mmus      AATWKNAVRHNLSLHKCFVRVENVKGAVWTVDEVEFQKRRPQKISGNPSLIKNMQSSHAY 593
FoxP2_ggor      AATWKNAVRHNLSLHKCFVRVENVKGAVWTVDEVEYQKRRSQKITGSPTLVKNIPTSLGY 602
FoxP2_mmul      AATWKNAVRHNLSLHKCFVRVENVKGAVWTVDEVEYQKRRSQKITGSPTLVKNIPTSLGY 603
Foxp2_mmus      AATWKNAVRHNLSLHKCFVRVENVKGAVWTVDEVEYQKRRSQKITGSPTLVKNIPTSLGY 603
FoxP2_ppyg      AATWKNAVRHNLSLHKCFVRVENVKGAVWTVDEVEYQKRRSQKITGSPTLVKNIPTSLGY 602
FoxP2_ptro      AATWKNAVRHNLSLHKCFVRVENVKGAVWTVDEVEYQKRRSQKITGSPTLVKNIPTSLGY 605
FOXP2_hsap      AATWKNAVRHNLSLHKCFVRVENVKGAVWTVDEVEYQKRRSQKITGSPTLVKNIPTSLGY 604
FoxP2_tgut      AATWKNAVRHNLSLHKCFVRVENVKGAVWTVDEVEYQKRRSQKITGSPTLVKNIPTSLGY 598
FOXP4_hsap      TATWKNAVRHNLSLHKCFVRVENVKGAVWTVDEREYQKRRPPKMTGSPTLVKNMISGLSY 554

FOXP1_hsap      CTPLNAALQASMAENSIPLYTTASMGNP-TLGNLASAIREELNGAMEHTNSNESDSSPGR 624
Foxp1_mmus      CTPLNAALQASMAENSIPLYTTASMGNP-TLGSLASAIREELNGAMEHTNSNESDSSPGR 652
FoxP2_ggor      GAALNASLQAALAESSLPLLSNPGLINN-ASSGLLQAVHEDLNGSLDHIDSN-GNSSPGC 660
FoxP2_mmul      GAALNASLQAALAESSLPLLSNPGLINN-ASSGLLQAVHEDLNGSLDHIDSN-GNSSPGC 661
Foxp2_mmus      GAALNASLQAALAESSLPLLSNPGLINN-ASSGLLQAVHEDLNGSLDHIDSN-GNSSPGC 661
FoxP2_ppyg      GAALNASLQAALAESSLPLLSNPGLINN-ASSGLLQAVHEDLNGSLDHIDSN-GNSSPGC 660
FoxP2_ptro      GAALNASLQAALAESSLPLLSNPGLINN-ASSGLLQAVHEDLNGSLDHIDSN-GNSSPGC 663
FOXP2_hsap      GAALNASLQAALAESSLPLLSNPGLINN-ASSGLLQAVHEDLNGSLDHIDSN-GNSSPGC 662
FoxP2_tgut      GAALNASLQAALAESSLPLLSNPGLINN-ASSGLLQAVHEDLNGSLDHIDSN-GNSSPGC 656
FOXP4_hsap      G-ALNASYQAALAESSFPLLNSPGMLNPGSASSLLPLSHDDVGAPVEPLPSNGSSSPPRL 613

FOXP1_hsap      SPMQAVHPVHVKEEPLDPEEAEGPLSLVTTANHSP-DFDHDRDYEDEPVNEDME 677
Foxp1_mmus      SPMQAVHPIHVKEEPLDPEEAEGPLSLVTTANHSP-DFDHDRDYEDEPVNEDME 705
FoxP2_ggor      SPQPHIHSIHVKEEPVIAEDEDCPMSLVTTANHSP-ELEDDREIEEEPLSEDLE 713
FoxP2_mmul      SPQPHIHSIHVKEEPVIAEDEDCPMSLVTTANHSP-ELEDDREIEEEPLSEDLE 714
Foxp2_mmus      SPQPHIHSIHVKEEPVIAEDEDCPMSLVTTANHSP-ELEDDREIEEEPLSEDLE 714
FoxP2_ppyg      SPQPHIHSIHVKEEPVIAEDEDCPMSLVTTANHSP-ELEDDREIEEEPLSEDLE 713
FoxP2_ptro      SPQPHIHSIHVKEEPVIAEDEDCPMSLVTTANHSP-ELEDDREIEEEPLSEDLE 716
FOXP2_hsap      SPQPHIHSIHVKEEPVIAEDEDCPMSLVTTANHSP-ELEDDREIEEEPLSEDLE 715
FoxP2_tgut      SPQPHIHSIHVKEEPVIAEDEDCPMSLVTTANHSP-ELEDDREIEEEPLSEDLE 709
FOXP4_hsap      SPPQYSHQVQVKEEPAEAEEDRQPGPPLGAPNPSASGPPEDRDLEEELPGEELS 667
```

References

1. Pani L, Overdier DG, Porcella A, Qian X, Lai E, Costa RH: **Hepatocyte nuclear factor 3β contains two transcriptional activation domains, one of which is novel and conserved with the *Drosophila* fork head protein.** *Mol Cell Biol* 1992, **12**(9):3723-3732.

2. Qian X, Costa RH: **Analysis of hepatocyte nuclear factor-3β protein domains required for transcriptional activation and nuclear targeting.** *Nucleic Acids Res* 1995, **23**(7):1184-1191.

3. Lai E, Prezioso VR, Tao WF, Chen WS, Darnell JE, Jr.: **Hepatocyte nuclear factor 3 alpha belongs to a gene family in mammals that is homologous to the *Drosophila* homeotic gene *fork head*.** *Genes Dev* 1991, **5**(3):416-427.

4. Copley RR: **The EH1 motif in metazoan transcription factors**. *BMC Genomics* 2005, **6**:169.

5. Yaklichkin S, Vekker A, Stayrook S, Lewis M, Kessler DS: **Prevalence of the EH1 Groucho interaction motif in the metazoan Fox family of transcriptional regulators**. *BMC Genomics* 2007, **8**:201.

6. Sullivan SA, Akers L, Moody SA: **foxD5a, a Xenopus winged helix gene, maintains an immature neural ectoderm via transcriptional repression that is dependent on the C-terminal domain**. *Dev Biol* 2001, **232**(2):439-457.

7. Overdier DG, Ye H, Peterson RS, Clevidence DE, Costa RH: **The winged helix transcriptional activator HFH-3 is expressed in the distal tubules of embryonic and adult mouse kidney**. *J Biol Chem* 1997, **272**(21):13725-13730.

8. Zhao X, Gan L, Pan H, Kan D, Majeski M, Adam SA, Unterman TG: **Multiple elements regulate nuclear/cytoplasmic shuttling of FOXO1: characterization of phosphorylation- and 14-3-3-dependent and -independent mechanisms**. *Biochem J* 2004, **378**(Pt 3):839-849.

9. Brownawell AM, Kops GJ, Macara IG, Burgering BM: **Inhibition of nuclear import by protein kinase B (Akt) regulates the subcellular distribution and activity of the forkhead transcription factor AFX**. *Mol Cell Biol* 2001, **21**(10):3534-3546.

10. Brunet A, Kanai F, Stehn J, Xu J, Sarbassova D, Frangioni JV, Dalal SN, DeCaprio JA, Greenberg ME, Yaffe MB: **14-3-3 transits to the nucleus and participates in dynamic nucleocytoplasmic transport**. *J Cell Biol* 2002, **156**(5):817-828.

11. Biggs WH, 3rd, Meisenhelder J, Hunter T, Cavenee WK, Arden KC: **Protein kinase B/Akt-mediated phosphorylation promotes nuclear exclusion of the winged helix transcription factor FKHR1**. *Proc Natl Acad Sci U S A* 1999, **96**(13):7421-7426.

12. Zhang X, Gan L, Pan H, Guo S, He X, Olson ST, Mesecar A, Adam S, Unterman TG: **Phosphorylation of serine 256 suppresses transactivation by FKHR (FOXO1) by multiple mechanisms. Direct and indirect effects on nuclear/cytoplasmic shuttling and DNA binding**. *J Biol Chem* 2002, **277**(47):45276-45284.

13. Woods YL, Rena G, Morrice N, Barthel A, Becker W, Guo S, Unterman TG, Cohen P: **The kinase DYRK1A phosphorylates the transcription factor FKHR at Ser329 in vitro, a novel in vivo phosphorylation site**. *Biochem J* 2001, **355**(Pt 3):597-607.

14. Nakae J, Park BC, Accili D: **Insulin stimulates phosphorylation of the forkhead transcription factor FKHR on serine 253 through a Wortmannin-sensitive pathway**. *J Biol Chem* 1999, **274**(23):15982-15985.

15. Rena G, Guo S, Cichy SC, Unterman TG, Cohen P: **Phosphorylation of the transcription factor forkhead family member FKHR by protein kinase B**. *J Biol Chem* 1999, **274**(24):17179-17183.

16. Brunet A, Bonni A, Zigmond MJ, Lin MZ, Juo P, Hu LS, Anderson MJ, Arden KC, Blenis J, Greenberg ME: **Akt promotes cell survival by phosphorylating and inhibiting a Forkhead transcription factor**. *Cell* 1999, **96**(6):857-868.

17. Brunet A, Park J, Tran H, Hu LS, Hemmings BA, Greenberg ME: **Protein kinase SGK mediates survival signals by phosphorylating the forkhead transcription factor FKHRL1 (FOXO3a)**. *Mol Cell Biol* 2001, **21**(3):952-965.

18. Kops GJ, de Ruiter ND, De Vries-Smits AM, Powell DR, Bos JL, Burgering BM: **Direct control of the Forkhead transcription factor AFX by protein kinase B**. *Nature* 1999, **398**(6728):630-634.

19. Takaishi H, Konishi H, Matsuzaki H, Ono Y, Shirai Y, Saito N, Kitamura T, Ogawa W, Kasuga M, Kikkawa U, Nishizuka Y: **Regulation of nuclear translocation of forkhead transcription factor AFX by protein kinase B**. *Proc Natl Acad Sci U S A* 1999, **96**(21):11836-11841.

20. Tang ED, Nunez G, Barr FG, Guan KL: **Negative regulation of the forkhead transcription factor FKHR by Akt**. *J Biol Chem* 1999, **274**(24):16741-16746.

21. Obsil T, Ghirlando R, Anderson DE, Hickman AB, Dyda F: **Two 14-3-3 binding motifs are required for stable association of Forkhead transcription factor FOXO4 with 14-3-3 proteins and inhibition of DNA binding**. *Biochemistry* 2003, **42**(51):15264-15272.

22. Rena G, Prescott AR, Guo S, Cohen P, Unterman TG: **Roles of the forkhead in rhabdomyosarcoma (FKHR) phosphorylation sites in regulating 14-3-3 binding, transactivation and nuclear targetting**. *Biochem J* 2001, **354**(Pt 3):605-612.

23. So CW, Cleary ML: **MLL-AFX requires the transcriptional effector domains of AFX to transform myeloid progenitors and transdominantly interfere with forkhead protein function**. *Mol Cell Biol* 2002, **22**(18):6542-6552.

24. Sublett JE, Jeon IS, Shapiro DN: **The alveolar rhabdomyosarcoma PAX3/FKHR fusion protein is a transcriptional activator**. *Oncogene* 1995, **11**(3):545-552.

25. Banham AH, Beasley N, Campo E, Fernandez PL, Fidler C, Gatter K, Jones M, Mason DY, Prime JE, Trougouboff P, Wood K, Cordell JL: **The FOXP1 winged helix transcription factor is a novel candidate tumor suppressor gene on chromosome 3p**. *Cancer Res* 2001, **61**(24):8820-8829.

26. Lai CS, Fisher SE, Hurst JA, Vargha-Khadem F, Monaco AP: **A forkhead-domain gene is mutated in a severe speech and language disorder**. *Nature* 2001, **413**(6855):519-523.

27. Shi C, Zhang X, Chen Z, Sulaiman K, Feinberg MW, Ballantyne CM, Jain MK, Simon DI: **Integrin engagement regulates monocyte differentiation through the forkhead transcription factor Foxp1**. *J Clin Invest* 2004, **114**(3):408-418.

28. Shu W, Yang H, Zhang L, Lu MM, Morrisey EE: **Characterization of a new subfamily of winged-helix/forkhead (Fox) genes that are expressed in the lung and act as transcriptional repressors**. *J Biol Chem* 2001, **276**(29):27488-27497.

29. Teufel A, Wong EA, Mukhopadhyay M, Malik N, Westphal H: **FoxP4, a novel forkhead transcription factor**. *Biochim Biophys Acta* 2003, **1627**(2-3):147-152.

30. Wang B, Lin D, Li C, Tucker P: **Multiple domains define the expression and regulatory properties of Foxp1 forkhead transcriptional repressors**. *J Biol Chem* 2003, **278**(27):24259-24268.

31. Li S, Weidenfeld J, Morrisey EE: **Transcriptional and DNA binding activity of the Foxp1/2/4 family is modulated by heterotypic and homotypic protein interactions**. *Mol Cell Biol* 2004, **24**(2):809-822.
